# Full-Photolithographic High-Density Skin-Like Transistor Arrays for All-Organic Active-Matrix Displays

**DOI:** 10.1007/s40820-026-02107-w

**Published:** 2026-03-04

**Authors:** Peng Xue, Juntong Li, Xiaoli Zhao, Yanping Ni, Hongyan Yu, Xianghui Liu, Bowen Xiang, Yao Fu, Junru Zhang, Baoying Sun, Pengbo Xi, Xiang Song, Yijun Shi, Guodong Zhao, Mingxin Zhang, Yanhong Tong, Qingxin Tang, Yichun Liu

**Affiliations:** Center for State Key Laboratory of Integrated Optoelectronics, and Key Lab of UV-Emitting Materials and Technology of Ministry of Education, College of Physics, Northeast Normal University, 5268 Renmin Street, Changchun, 130024 People’s Republic of China

**Keywords:** Active-Matrix, Organic thin-film transistors, Photolithography, Organic light-emitting diodes

## Abstract

Organic thin-film transistors (OTFTs) are widely recognized as promising building blocks for next-generation flexible and wearable electronics. However, scalable fabrication of high-density OTFT arrays for active-matrix applications remains highly challenging, primarily due to the incompatibility of conventional photolithography with organic semiconductors. Here, we report an all-photolithographic strategy that enables the scalable fabrication of flexible OTFT arrays with both high device density and superior charge transport characteristics. By combining synergistic interfacial modulation and dual-protection photolithography strategy of organic semiconductors, we successfully fabricated transistor arrays exhibiting an average mobility above 1.0 cm^2^ V^−1^ s^−1^ and on/off ratios of ~ 10^5^. This scalable method further enables an integration density of 6.25 × 10^4^ cm^−2^, which is one of the highest densities reported to date for full-photolithographic OTFT active-matrix arrays. Moreover, we demonstrate seamless integration of OTFT active-matrix arrays with organic light-emitting diodes (OLEDs), yielding all-organic active-matrix OLED (AMOLED) arrays. These devices exhibit stable electroluminescence, ultralight weight (~ 24.3 g m^−2^), excellent flexibility, and skin-like display functionality with reliable pixel-level addressing. This work establishes a universal and scalable route toward high-density organic electronic systems, opening new opportunities for flexible displays, electronic skin, and next-generation wearable technologies.

**Supplementary Information:**

The online version contains supplementary material available at 10.1007/s40820-026-02107-w.

## Highlights

- A universal and scalable process fully compatible with organic semiconductors is developed, achieving precise micropatterning and high integration density of 6.25 × 10^4^ cm^−2^.
- All-organic active-matrix organic light-emitting diode arrays with skin-like deformability were successfully fabricated. Notably, the integrated system not only exhibits stable electroluminescence, ultralight weight (~24.3 g m^−2^), and strong conformability to complex surfaces but also paves a promising path for the development of next-generation flexible and wearable displays.

**Supplementary Information:**

The online version contains supplementary material available at 10.1007/s40820-026-02107-w.

## Graphical abstract

**Figure:**
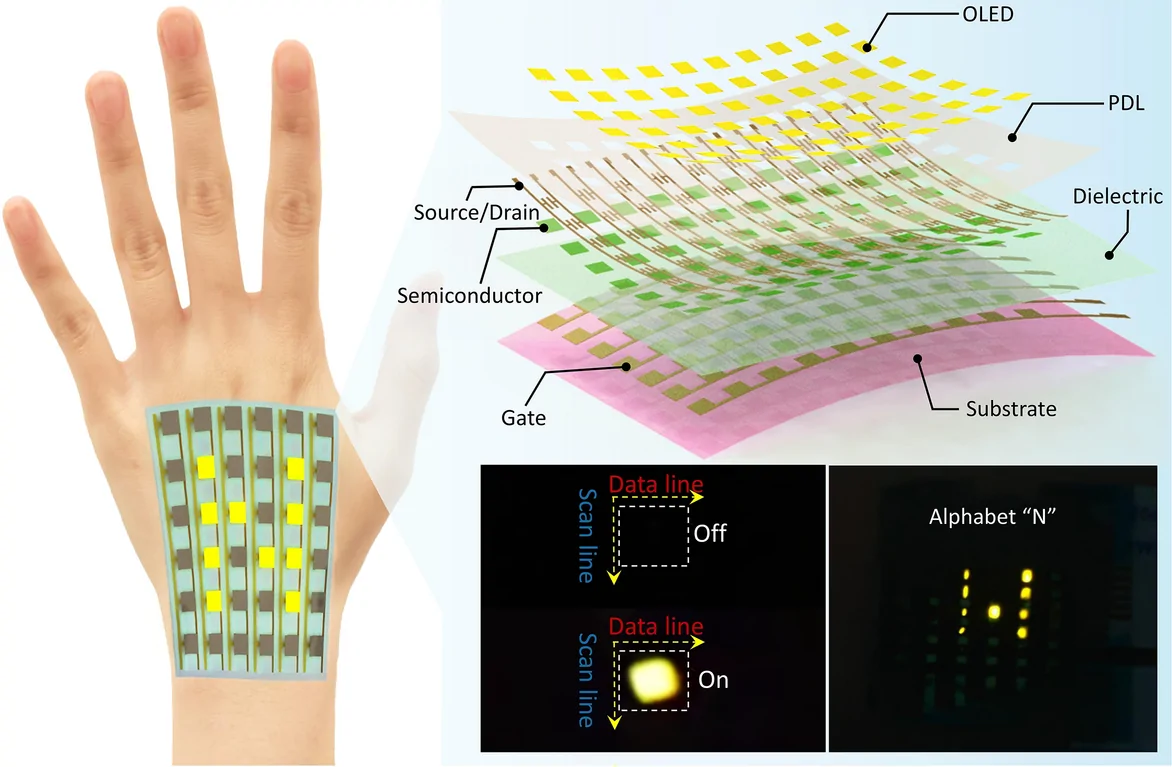

## Introduction

Organic thin-film transistor (OTFT) offers irreplaceable advantages for the development of next-generation flexible and elastic electronics, owing to its exceptional mechanical flexibility, low-temperature processability, and lightweight nature [1–6]. Notably, the high-density integration of OTFT arrays serves as a critical prerequisite for advancing the practical implementation of their functionalities [7]. As a core building block, high-density OTFT arrays not only underpin high-resolution flexible displays but also support emerging flexible electronics (e.g., electronic skin, wearable sensors) in delivering precise sensing and signal processing capabilities [8–11]. However, in the vast majority of currently reported OTFT arrays, the devices within the array still adopt an independently designed structure, lacking a unified shared lead design [12]. This structural limitation directly gives rise to two key issues: first, the design complexity of the array drive circuit is significantly increased, which raises the difficulty of drive control; second, the wiring density is greatly enhanced, leading to obvious wiring congestion in the array layout [13]. The combination of the above issues not only increases the manufacturing and integration costs of OTFT arrays, but also restricts their practical application and industrialization progress in high-performance systems such as high-resolution displays and large-scale flexible sensing. To overcome signal interference and ensure reliable operation, the development of OTFT active-matrix arrays with shared addressing leads is indispensable for high-resolution soft electronics. In this array architecture, each transistor preserves independent gate, source, and drain terminals that are directly connected to shared row and column lines, enabling individual electrical access to each array element. Such a configuration effectively suppresses array-level crosstalk, improves addressing efficiency, and supports high integration density [14–16].

At present, achieving OTFT arrays that simultaneously exhibit high integration density and superior device performance still faces formidable technological bottlenecks [17–19]. The realization of high-density integration in OTFT arrays essentially relies on the downscaling of device feature sizes and the minimization of interconnect pitch, while high performance is critically dependent on efficient carrier injection and transport [20–22]. However, when the array density is scaled up to several thousand or even tens of thousands of transistors per square centimeter, conventional fabrication techniques encounter inherent limitations, including insufficient patterning resolution, insufficient alignment accuracy, and inadequate uniformity and scalability, which ultimately hinder their ability to meet the stringent electrical requirements of high-resolution displays and precision electronic skins [23–25]. By contrast, photolithography, as the cornerstone technology of modern microelectronics and optoelectronics, offers unrivaled patterning resolution, exceptional interlayer alignment precision, and robust large-area integration capacity, thereby establishing itself as the indispensable fabrication technique for large-scale integration of high-density OTFT arrays [19, 26–29]. For instance, Goettling et al. demonstrated a 64× 64 active-matrix OTFT array backplane fabricated entirely by standard photolithographic processes, where Al_2_O_3_ gate dielectrics enabled low operating voltages. The resulting backplane was further integrated into a 20 mm × 20 mm polymer-dispersed liquid crystal (PDLC) display [30]. Similarly, Lee et al. reported an all-photolithographic flexible OTFT active-matrix array using a highly stable organic semiconductor, and further demonstrated its feasibility as an acetone vapor sensor [31]. While photolithography has demonstrated remarkable efficacy in advancing the large-scale integration of OTFT arrays, its intrinsic incompatibility with organic semiconductors remains a fundamental challenge. Owing to the high chemical sensitivity of organic semiconductors, solvents used in photolithography tend to induce interfacial damage and molecular degradation in the latter, which in turn significantly degrades charge transport efficiency [32, 33]. This issue poses a direct constraint on two critical fabrication processes: firstly, it is challenging to directly pattern the organic semiconductor layer for crosstalk suppression; secondly, it is not feasible to directly fabricate source-drain electrodes on the semiconductor surface via photolithography. Consequently, the intrinsic incompatibility between photolithography and organic semiconductors remains the challenge constraining the high-density integration of field-effect transistor arrays.

Here, we report a full-photolithographic fabrication strategy for constructing high-density and high-performance skin-like OTFT active-matrix arrays. By integrating “synergistic interfacial modulation” with “protective layer photolithography” techniques, we achieved arrays with broadly tunable integration densities. The maximum device density reaches 6.25 × 10^4^ cm^−2^, which is one of the highest values reported to date for full-photolithographic OTFT active-matrix arrays. Importantly, across all integration levels, the devices consistently exhibit an average carrier mobility exceeding 1 cm^2^ V^−1^ s^−1^. Moreover, the system-level integration capability of this strategy is demonstrated by directly integrating OTFT arrays with organic light-emitting diodes (OLEDs) to realize an addressable all-organic active-matrix OLED (AMOLED) array. This work establishes a versatile and scalable route to high-density, high-performance OTFT active-matrix arrays and underscores their promise for next-generation wearable and skin-like electronic systems.

## Experimental Section

### Materials

All processing materials were purchased from commercial sources and used as received. Organic semiconductors poly[[2,5-bis(2-octyldodecyl) − 2,3,5,6-tetrahydro-3,6-dioxopyrrolo[3,4-c] pyrrole-1,4-diyl]-alt-[[2,2’-(2,5-thiophene)bis-thieno(3,2-b)thio-phene] − 5,5’-diyl]] (DPPT-TT) and indacenodithiophene-benzothiadiazole (IDTBT, average Mw ≈ 340 kDa) were bought from Derthon Optoelectronic Materials Co. Ltd. Octadecyl-trichlorosilane (OTS, 95%) and pentafluorothiophenol (PFBT, 95%) were bought from Acros and TCI (Shanghai) Development Co. Ltd, respectively. Poly(3,4-ethylenedioxythiophene):poly(styrenesulfonate) (PEDOT:PSS, PH1000) was acquired from Heraeus. Organosilicone (DC 1–2577) was acquired from Dow Corning and was dissolved in OS-20 (Dow Corning Co.). Surfactants (Capstone FS-30) and ethylene glycol (EG) were provided by DuPont and Sigma-Aldrich, respectively. SEBS H1052 was purchased from the Asahi Kasei company. Poly(vinyl alcohol) PVA was obtained from Sigma-Aldrich. NOA68 was purchased from Norland Corporation. All small-molecule organic semiconductors used to prepare OLED devices were purchased from Xi’an Yuri Solar Co., Ltd.

### Fabrication of Micro-Patterned Organic Semiconductors

A dual protective layer approach was employed to achieve non-destructive photolithographic patterning of the organic semiconductor. First, PVA solution was spin-coated onto the IDTBT surface (6000 rpm, 40 s), followed by thermal annealing at 70 °C for 10 min to form an anti-solvent protection layer. Subsequently, 5 mg mL^−1^ DPPT-TT solution was spin-coated at 4000 rpm for 60 s as a moisture barrier layer, and the film was thermally annealed at 70 °C for 10 min to remove residual solvent. After the formation of the dual protection layers, a photoresist (AZ 5214E) was spin-coated on top and exposed to UV light to define the desired pattern. The unprotected regions were then etched by oxygen plasma, selectively removing the exposed areas of the semiconductor film. Finally, the photoresist and protection layers were sequentially removed using acetone, chloroform, and deionized water, yielding a well-defined micropatterned semiconductor thin film.

### Fabrication of Skin-Like OTFT Active-Matrix Array

Fabrication of the skin-like OTFT array: i) The patterned source-drain electrodes and gate electrodes (30 nm Au) were prepared on OTS-modified Si wafers by a lift-off photolithography process. ii) Soaked the Au source-drain electrodes in PFBT solution (PFBT: toluene = 1:1000 by volume) for 2 min. iii) Organic semiconductor IDTBT (5 mg mL^−1^, solvent is trichloromethane) was spin-coated on source-drain electrodes (1500 rpm, 60 s) and then annealed in nitrogen for 30 min at a temperature of 100 °C . The semiconductor was patterned using the aforementioned deposition method. iv) Organosilicone DC 1–2577 was dissolved in OS-20 with a volume ratio of 1:4. The configured solution was spin-coated on IDTBT (5000 rpm, 60 s) and heated at 70 °C for 30 min to attain a uniform film, which served as the gate dielectric. The measured capacitance (*C*_i_) is 3.3 nF cm^−2^. v) Dielectric was exposed in a plasma oxidation chamber (100 W, 120 s), aligned with the gate electrode embedded in NOA68, and then heated in an oven at 70 °C for 30 min. vi) The skin-like OTFT array was obtained by stripping the overall structure from the OTS/Si substrates.

### Fabrication of Skin-Like AMOLED Array

First, the OTFT active-matrix arrays were fabricated following the same procedures described above. The source and drain electrodes were composed of PEDOT:PSS, which were micropatterned via the dual protective layer photolithography technique. Subsequently, the organic semiconductor IDTBT was spin-coated onto the patterned electrodes (1500 rpm, 60 s) and annealed at 100 °C for 30 min in nitrogen. Unlike the previous array fabrication, the semiconductor layer was not patterned in this process. Afterwards, in accordance with the same procedure described in Sect. 2.3, DC 1–2577 were spin-coated to fabricate the OTFT active-matrix array. Meanwhile, on the OTS/Si substrate, patterned SEBS was fabricated via the double protective layer method and used as the pixel-defining layer. Subsequently, the fabricated OTFT array was precisely aligned and laminated with the patterned SEBS, and the entire structure was peeled off from the OTS/Si. Finally, the OLED functional layers were sequentially deposited on the PDL-defined regions. This process yielded an all-organic AMOLED device.

## Results and Discussion

### Device Design and Fabrication

**Fig. 1:**
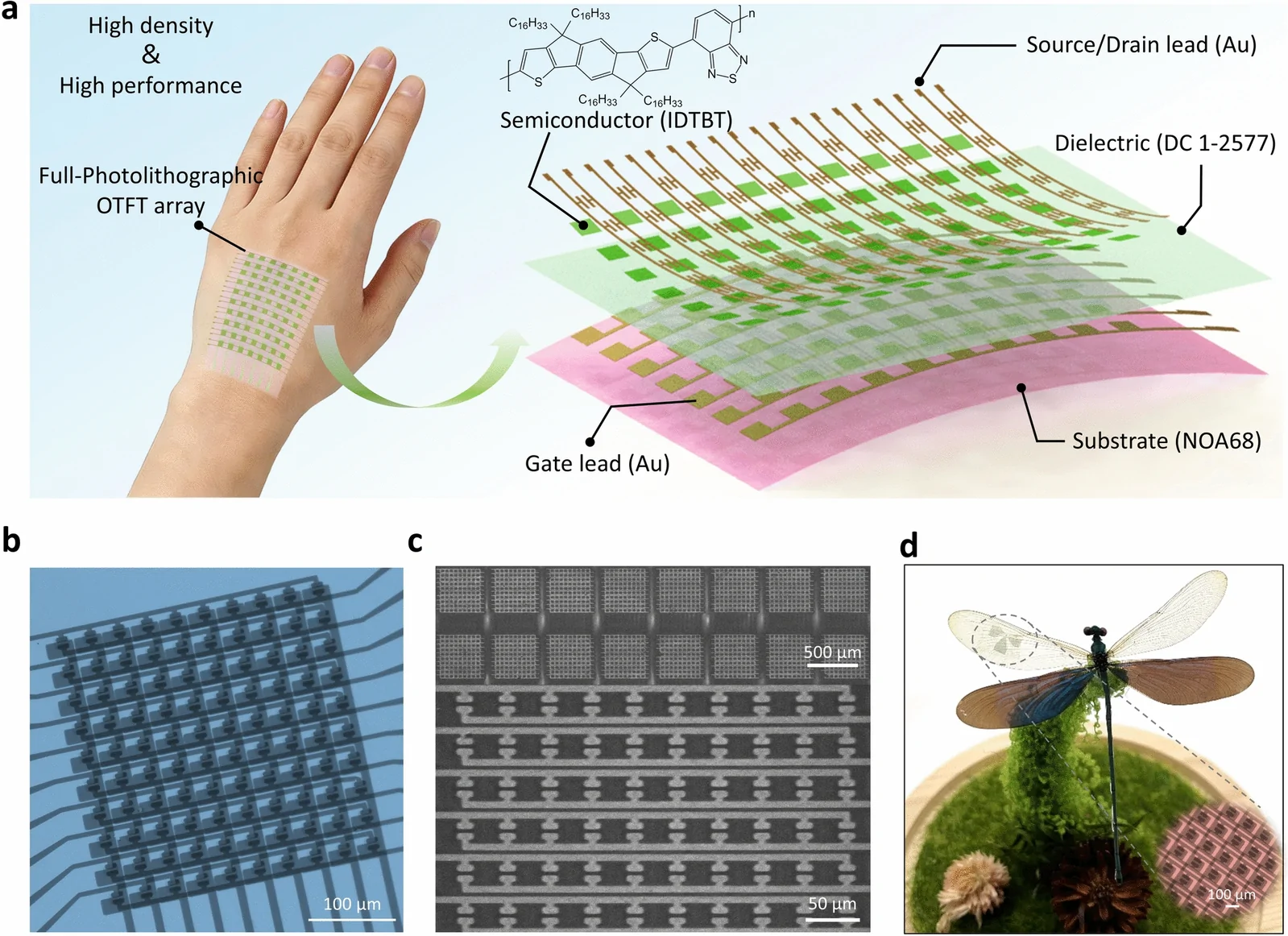
Design and fabrication of full-photolithographic skin-like OTFT active-matrix arrays. **a** Schematic illustration showing the essential requirements of OTFT active-matrix arrays for practical flexible and skin-like electronic applications. The right part depicts the multilayer device structure comprising substrate, gate leads, dielectric, semiconductor, and source/drain leads. **b** Optical micrograph of the OTFT active-matrix array, highlighting the row/column addressing configuration. **c** SEM image showing micron-scale precision of electrode and interconnect features. **d** Photograph of a flexible OTFT array conformably attached to the surface of a dragonfly wing, demonstrating its ultralight and skin-like nature

To realize core functionalities required for skin-like electronic devices, such as multimodal sensing, signal processing, and driving regulation, the construction of high-density, high-performance, and skin-like OTFT arrays is a key prerequisite (Fig. 1a). Skin-like electronics must adapt to the complex curvatures figure of the human body and support precise applications through multi-dimensional signal acquisition with high spatial resolution, which imposes stringent requirements on the conformability, integration density, and electrical performance of the devices [34, 35]. Although substantial progress has been made in flexible electronics through material innovations and process optimizations, the fabrication of OTFT arrays that simultaneously achieve high integration density and excellent electrical performance still lacks a universally applicable and precisely controllable process, which hinders the advancement of skin-like electronics toward high-resolution, multifunctional integration. To address the above requirements and challenges, we propose a skin-like device fabrication strategy based on a full-photolithography process, in which a system-level co-design strategy is adopted by jointly optimizing nondestructive photolithographic patterning of organic semiconductors, device architecture, and electrode–semiconductor interfaces, thereby enabling reliable electrical performance and mechanical robustness on flexible substrates.

To ensure the high-performance OTFT array fabricated via the full- photolithography process in this study, we carried out the optimization design from the following aspects: 1) Optimization of device structure and source-drain electrodes. In conventional top-contact devices, source-drain electrodes are mostly fabricated by direct deposition on the semiconductor surface. Since this approach tends to damage organic semiconductors and exhibits poor compatibility with the photolithography process, it poses obstacles to the high-density integration of arrays. To address this issue, we adopted an “inverted bottom-gate top-contact architecture” fabrication strategy: high-resolution Au source-drain electrode patterns were pre-patterned on a rigid substrate using photolithography, followed by the deposition of the semiconductor layer. This fundamentally eliminates damage to organic semiconductors during electrode fabrication. Meanwhile, the embedded contact formed between the source-drain electrodes and the semiconductor offers a larger charge injection area compared to bottom-gate bottom-contact structures, which is conducive to enhancing carrier mobility (Fig. S1). The Au electrodes not only possess an energy level matching that of organic semiconductors (capable of reducing the carrier injection barrier) and high electrical conductivity (minimizing the lead resistance in array interconnections), but also their surfaces, after modification with pentafluorobenzenethiol (PFBT), can further reduce the injection barrier through work function modulation, thereby optimizing the interfacial contact resistance [36, 37]. 2) Non-destructive patterning of organic semiconductors. The non-destructive and micro-scale precise patterning of organic semiconductors was achieved via the dual-protective photolithography technique. Figure S2 demonstrates that, unlike unprotected samples, organic semiconductors processed with the dual-protection strategy retain their original morphology and transistor performance, confirming the nondestructive nature of the proposed patterning technique. This method can effectively reduce the device leakage current (Fig. S3), improve the electrical performance uniformity under high-density integration, and mitigate crosstalk between adjacent devices (Fig. S4), thus providing a guarantee for the stability of array performance. Figure S5 compares existing photolithographic methods for organic semiconductors with the dual-protection photolithographic strategy, demonstrating the latter’s superior performance in organic semiconductor patterning. 3) Optimization of interlayer contact. Organosiloxane DC 1–2577 was selected as the dielectric layer material, which combines excellent dielectric properties and flexibility. Moreover, its diluent (OS-20) is non-destructive to organic semiconductors, effectively resolving the compatibility challenge between the solution-processed dielectric layer and the semiconductor layer. Furthermore, surface hydroxylation treatment was performed on the DC 1–2577 dielectric layer, which enhances the interfacial interaction between the dielectric layer and the gate electrode. This not only improves the structural integrity of the device during peeling from the rigid substrate (suppressing interlayer delamination) but also further optimizes the device performance. 4) Adaptive design of gate electrodes for high-density integration. The photocurable polymer NOA68 was used as the substrate material for gate electrodes. It exhibits strong interfacial adhesion with Au electrodes, and the “embedded” electrode structure formed after NOA68 curing ensures that the Au electrodes maintain structural integrity and electrical conductivity stability during substrate transfer and bending deformation [38]. Meanwhile, the photocurable property of NOA68 is compatible with the photolithography process, enabling micro-scale precise patterning of gate electrodes and thus laying a critical foundation for the construction of high-density arrays.

Based on the comprehensive optimization design, we can establish a universal full-photolithography fabrication strategy, successfully realizing the fabrication of an OTFT active-matrix array with both high performance and high density (see Fig. S6 for the detailed fabrication process). This strategy is compatible with a variety of materials. In terms of array design, we adopted a “shared lead” structure at the source, drain, and gate terminals (Fig. 1b). This interconnection method significantly reduces wiring complexity while ensuring the electrical independence of individual devices, providing a new design concept for the scalable fabrication of large-area flexible electronic circuits. Scanning electron microscopy (SEM) images (Fig. 1c) further confirm that the feature sizes of the electrodes and leads in the array reach the micron scale, and the pattern boundaries are clearly defined. This observation verifies the precision of the full-photolithography process. Notably, the array exhibits excellent flexibility and lightweight characteristics, enabling it to seamlessly conform to ultra-light flexible surfaces such as dragonfly wings (Fig. 1d). This fully demonstrates its adaptation potential in scenarios including wearable systems and flexible displays.

### High-Density OTFT Active-Matrix Arrays

**Fig. 2:**
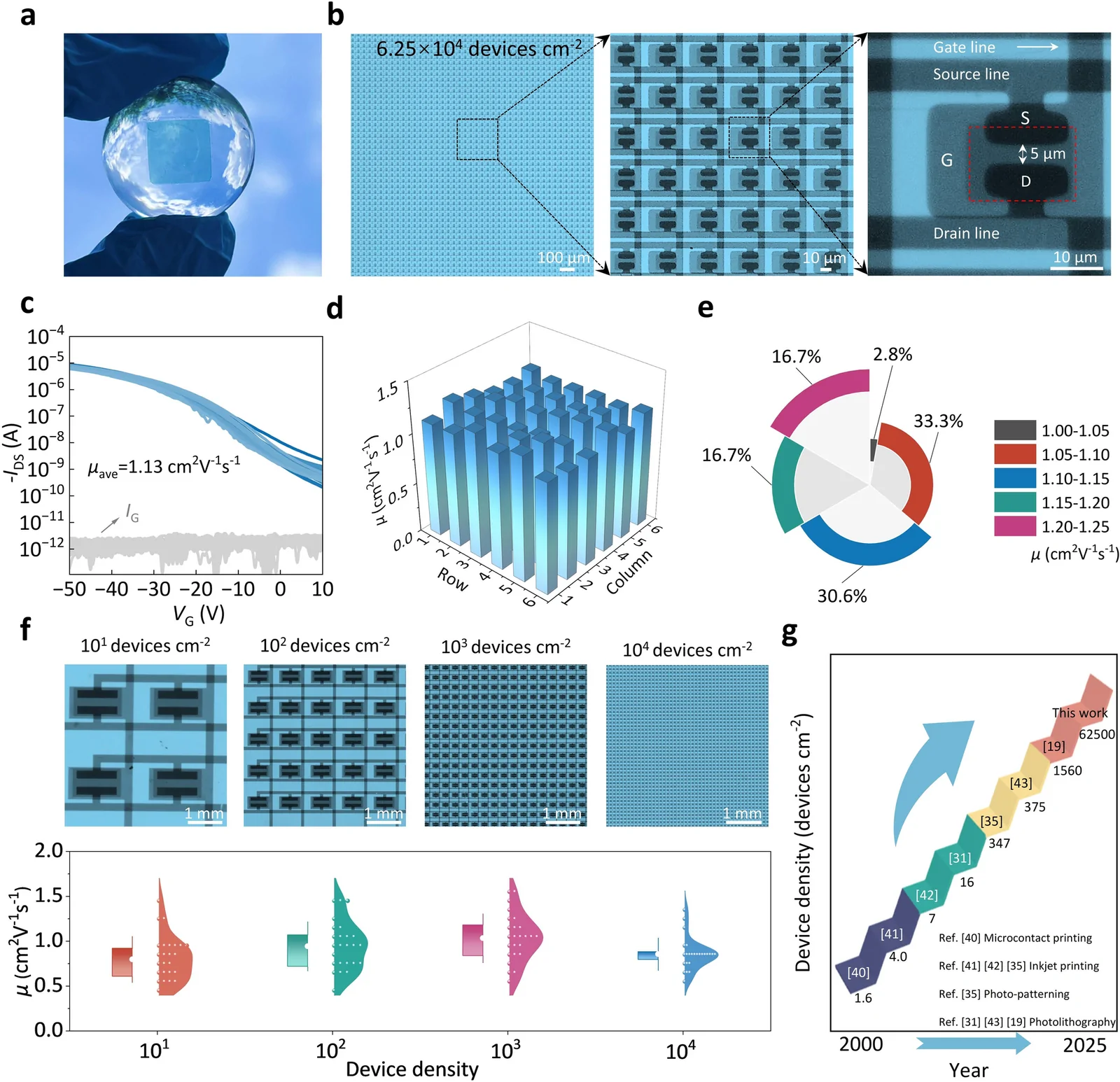
High-density and uniform performance of full-photolithographic OTFT arrays. **a** Photograph of a flexible OTFT array conformally attached to a 3D spherical surface. **b** Optical microscopy images at different magnifications showing highly ordered transistor arrangements and clearly defined semiconductor/electrode. **c** Transfer characteristics of 36 randomly selected devices, showing low leakage currents and *p*-type behavior. **d** Three-dimensional distribution map of carrier mobility across the array, showing uniform mobility with an average of 1.13 cm^2^ V^−1^ s^−1^. **e** Statistical analysis of mobility distribution within the array. **f** Optical images of arrays with different integration densities (10^1^–10^4^ cm^−2^) and their corresponding electrical performance. **g** Comparison of integration density in this work with previously reported OTFT active-matrix arrays, showing a high integration level

High-density and scalable device integration capability serves as the core enabler for translating OTFT arrays from laboratory research to practical applications [39]. To further verify the scalability and universality of the integration strategy proposed in this study, breakthrough progress in device density and performance is demonstrated in Fig. 2. Figure 2a presents an optical image of the OTFT array conformed to a three-dimensional spherical surface, intuitively verifying the excellent mechanical flexibility of the devices, which are critical prerequisites for the realization of flexible electronic applications. Optical microscopy images at different magnifications clearly reveal the compact and highly ordered arrangement of the transistor array, as well as the distinct interfaces between the source/drain electrodes and the micropatterned semiconductor regions (Fig. 2b). Benefiting from the high-resolution advantage of photolithography technology, the minimum channel length is precisely reduced to 5 μm. It should be noted that this only represents the technical limit under the current photolithography process conditions, and the device integration density is expected to be further increased to over 10^5^ cm^−2^ in the future by further reducing the channel length and electrode width.

As shown in Fig. 2c, the transfer characteristics of 36 randomly selected devices from the array exhibit typical *p*-type behavior. Benefiting from the precise micropatterning of the semiconductor layer, the transistors display extremely low leakage currents. The three-dimensional distribution of carrier mobility is presented in Fig. 2d, demonstrating that, despite the significantly increased device density, the transistors maintain high and uniform mobilities across the entire array with an average value of 1.13 cm^2^ V^−1^ s^−1^. The statistical analysis in Fig. 2e further reveals that all devices exhibit mobilities above 1 cm^2^ V^−1^ s^−1^, with more than 60% exceeding 1.10 cm^2^ V^−1^ s^−1^, highlighting the stability and reliability of the fabrication process. In addition, mechanical stability is a crucial metric for evaluating the performance of skin-like transistors. Therefore, the evolution of device mobility under cyclic bending and the strain-dependent electrical characteristics were systematically investigated. As shown in Fig. S7a, the devices were subjected to repeated folding cycles at a bending radius of 1 mm. Even after 500 folding cycles, the normalized mobility (*μ*/*μ*_0_) remains above 90%, indicating excellent mechanical durability under severe deformation conditions relevant to skin-like applications. Furthermore, strain-dependent electrical characteristics were examined over a range of bending radii from 3 to 1 cm, corresponding to calculated tensile strains of approximately 0.07%–0.20% (Fig. S7b). Across the entire strain range, key electrical parameters, including charge carrier mobility, on-state current, and off-state current, show negligible variation, demonstrating stable electrical performance under mechanical bending. Collectively, these results confirm the outstanding mechanical robustness of the skin-like transistors against both folding and bending deformations.

Equally important, this strategy ensures excellent device uniformity across a broad range of integration densities. The upper panel of Fig. 2f shows optical images of arrays with varying densities, illustrating the transition from low to high integration while maintaining device fidelity. Across a wide density range (10^1^ – 10^4^ cm^−2^), the mobility distribution remains nearly unchanged, consistently falling within 0.50 – 1.50 cm^2^ V^−1^ s^−1^. This result underscores the robustness of our approach and represents a critical step toward bridging laboratory-scale demonstrations with practical large-area manufacturing of organic electronics. Comparison with previously reported results confirms that our achieved density of 6.25 × 10^4^ cm^−2^ represents one of the highest integration level among OTFT active-matrix arrays reported to date (Fig. 2g and Table S1) [19, 31, 35, 40–43]. Overall, through the in-depth integration of full-photolithography processes and flexible electronic materials, this study resolves the core contradiction between “high-density integration” and “high-performance synergy”, laying a technical foundation for the practical application of future skin-like electronics and flexible intelligent systems.

### Synergistic Interfacial Modulation

**Fig. 3:**
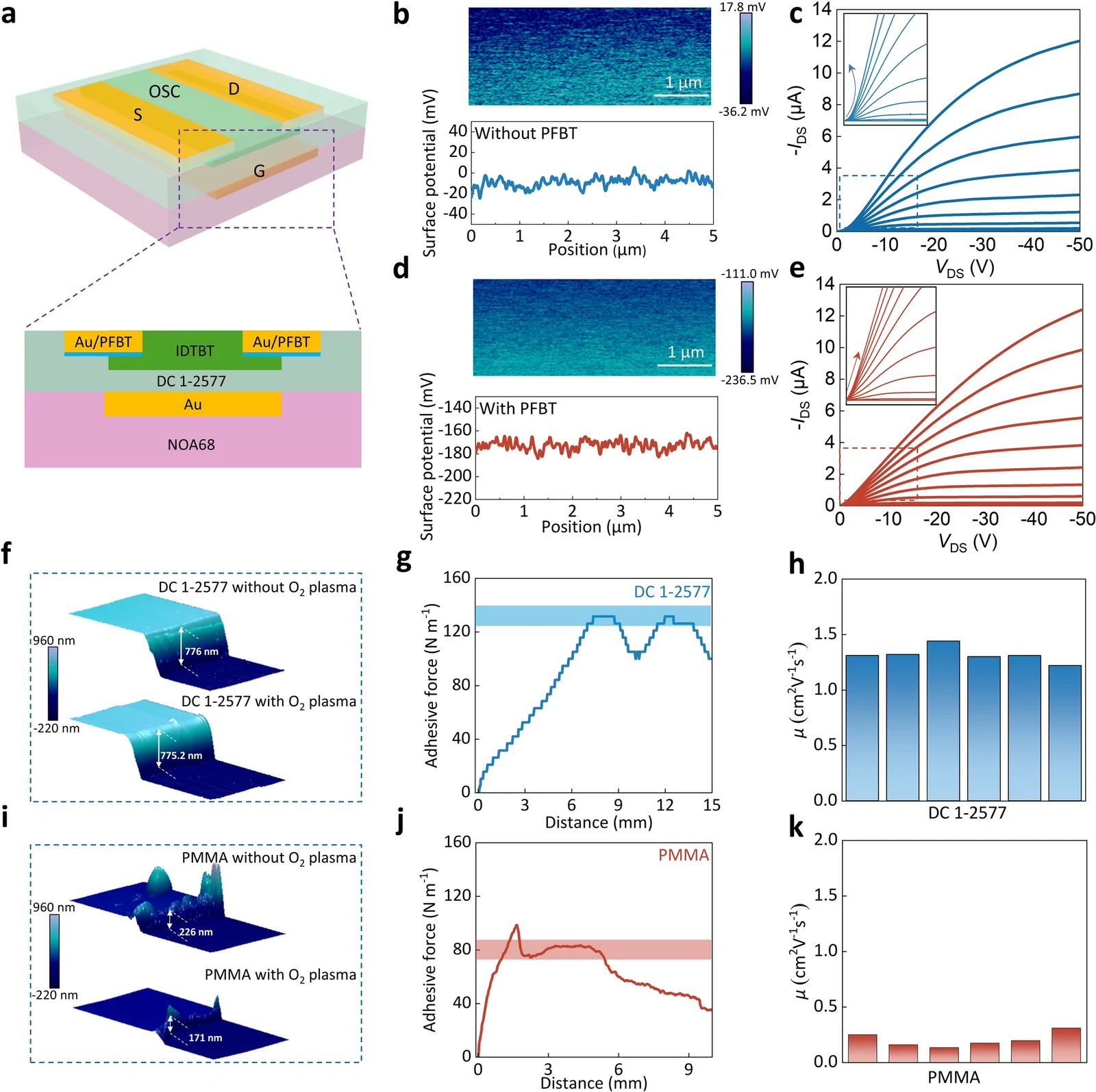
Synergistic interfacial modulation enabling efficient charge injection and transport. **a** Schematic structure of the OTFT device comprising PFBT-modified Au electrodes, IDTBT semiconductor, and organosiloxane dielectric. **b** KPFM measurements of Au electrodes without PFBT modification. **c** Output characteristics of OTFTs using unmodified Au electrodes, exhibiting nonlinear *I*_DS_–*V*_DS_ behavior due to high contact resistance. **d** KPFM measurements of Au electrodes with PFBT modification. **e** Output characteristics of OTFTs with PFBT-modified electrodes, displaying steeper slopes, indicative of improved charge injection. **f** Thickness of DC 1–2577 dielectric before and after oxygen-plasma treatment. **g** Adhesion-force curve between the plasma-treated DC 1–2577 layer and Au/NOA68 gate electrode. **h** Carrier mobility distribution of devices using DC 1–2577 dielectric. **i** Thickness of PMMA dielectric before and after oxygen-plasma treatment. **j** Adhesion-force curve between PMMA and Au/NOA68 gate electrode. **k** Carrier mobility distribution of devices using PMMA dielectric

To elucidate the physical mechanism underlying the high performance achieved by the proposed strategy, a systematic investigation was conducted from two perspectives: carrier injection and transport. Figure 3a illustrates the schematic device configuration, clearly presenting the stacked structure consisting of self-assembled monolayer (SAM)-modified Au electrodes, the organic semiconductor (IDTBT), and the dielectric layer. Figure 3b–e further examines the interfacial properties of the Au electrodes and their influence on the electrical characteristics of the device. The surface potential of the electrodes was characterized using Kelvin probe force microscopy (KPFM). For the Au electrode without PFBT modification, the measured surface potential was approximately -0.009 V (Fig. 3b), corresponding to a work function of about 5.11 eV. After PFBT modification, the surface potential decreased markedly to approximately -0.173 V (Fig. 3d), resulting in an enhanced work function of 5.28 eV. The increased work function effectively optimizes the energy level alignment between Au and IDTBT (~ 5.4 eV) [44], thereby reducing the hole injection barrier. Consistently, the output characteristics of the devices show more linear *I*_DS_–*V*_DS_ curves with steeper slopes and higher saturation currents (Fig. 3c, e), indicating a significant reduction in contact resistance and an improvement in charge injection efficiency. Quantitatively, this reduction in contact resistance is corroborated by transmission line method (TLM) measurements displayed in Fig. S8. The contact resistance of the devices is reduced from 90 kΩ cm for the unmodified Au electrodes to 36 kΩ cm after PFBT modification.

Beyond interfacial optimization at the injection side, the contact quality between the dielectric layer and the gate electrode plays a crucial role in ensuring conformal interfacial contact and efficient electrostatic modulation, thereby enabling effective carrier accumulation and transport in the channel. Two dielectric materials compatible with the photolithographic process, DC 1–2577 and polymethyl methacrylate (PMMA), were selected for comparison (Fig. 3f – k). DC 1–2577 and PMMA employ silicone oil and butyl acetate as solvents, respectively, where silicone oil causes no chemical degradation to organic semiconductors, while butyl acetate acts as an orthogonal solvent. Benefiting from the siloxane backbone, DC 1–2577 can be subjected to oxygen plasma treatment to induce surface hydroxylation, which strengthens its interfacial adhesion with the gate electrode. As shown in Fig. 3f, the thickness of DC 1–2577 before and after plasma treatment was 776 and 775.2 nm, respectively, remaining essentially unchanged. Adhesion force measurements further confirm the enhanced interfacial interaction between the treated DC 1–2577 layer and the gate electrode (Fig. 3g), providing both mechanical robustness and electrical stability to the device. In contrast, the PMMA dielectric layer exhibits severe etching and surface roughening after oxygen plasma exposure (Figs. 3i and S9), leading to a pronounced reduction in thickness (from 226 to 171 nm) and a nearly fivefold increase in surface roughness (from 0.246 to 1.15 nm). Consequently, oxygen plasma treatment cannot be applied to PMMA-based dielectrics, and the interfacial adhesion with the gate electrode remains weak (Fig. 3j), which deteriorates carrier transport and overall device performance. Quantitative mobility analysis (Fig. 3h, k) demonstrates that devices using DC 1–2577 retain high and stable electrical performance, whereas those employing PMMA exhibit a substantial decrease in mobility. Notably, when comparing laminated-gate devices with devices in which the gate electrode was directly evaporated onto the dielectric layer, PMMA-based devices show higher mobilities in the directly evaporated configuration, indicating the presence of physical gaps or imperfect interfacial contact in laminated-gate devices that deteriorate charge transport. In contrast, DC 1–2577-based devices exhibit nearly identical mobilities for both laminated and evaporated gate configurations, confirming that the oxygen-plasma-treated DC 1–2577 dielectric forms intimate and robust contact with the gate electrode without compromising device performance (Fig. S10).

All in all, the PFBT-modified Au electrodes facilitate efficient charge injection by lowering the interfacial barrier, while the plasma-treatable organosiloxane dielectric layer ensures smooth and stable carrier transport. Importantly, comparative control experiments reveal that PFBT modification leads to only marginal performance improvement when PMMA is used as the dielectric, whereas a much more pronounced mobility enhancement is observed when PFBT is combined with DC 1–2577. These results strongly support a synergistic interfacial modulation effect arising from the combined optimization of electrode modification and dielectric engineering, rather than from either factor alone (Fig. S11). The synergistic coupling of these two interfacial optimizations therefore constructs a unified electrode–semiconductor–dielectric system characterized by low barrier height, strong interfacial adhesion, and superior operational stability, which collectively underpin the high performance of the developed devices. It is worth noting that by adopting thinner or higher-k dielectrics, low-voltage operation suitable for flexible electronic skin applications is promising to be achieved in future studies.

### All-Organic AMOLED Arrays

**Fig. 4:**
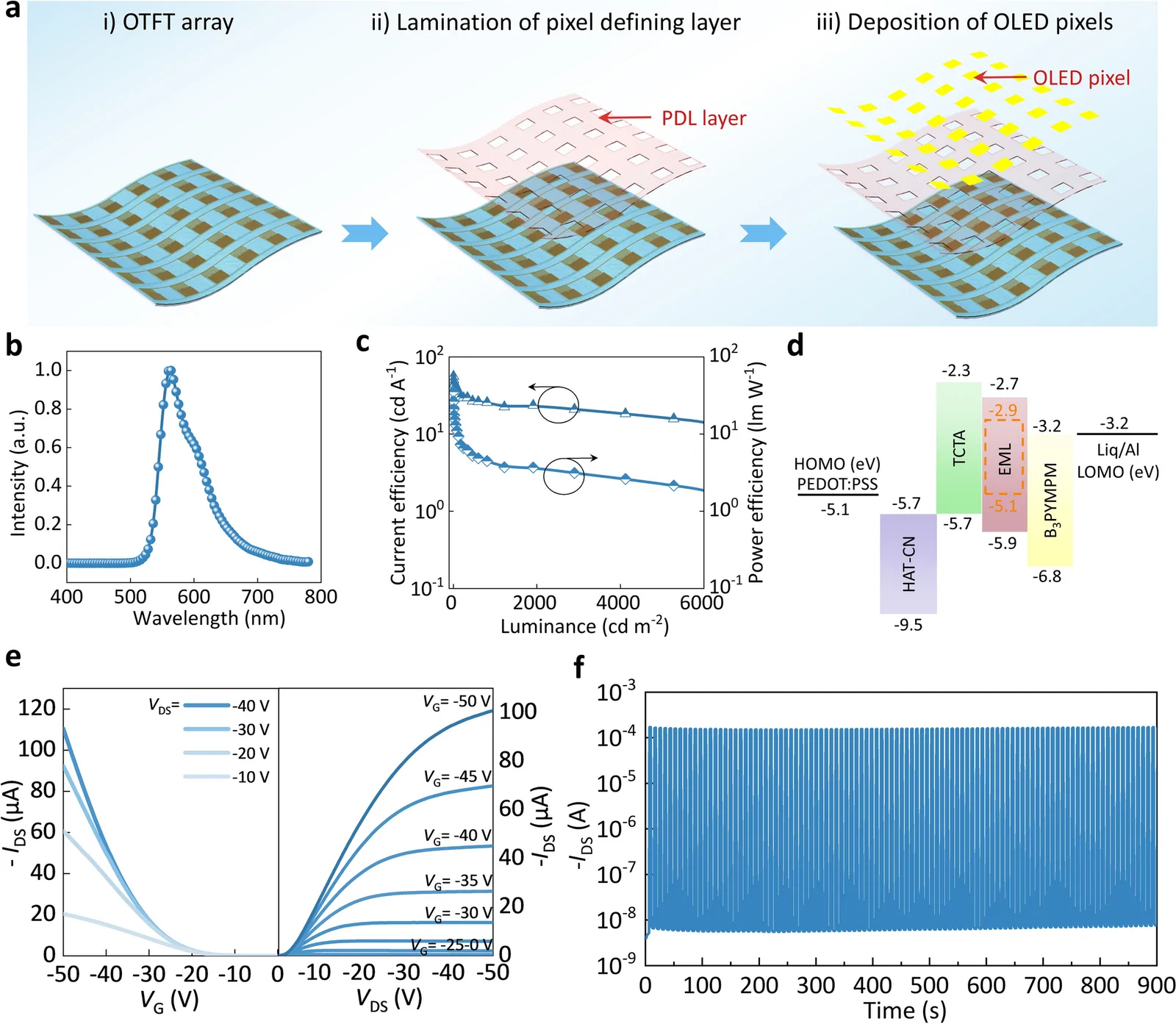
Fabrication and characterization of all-organic AMOLED devices driven by OTFT arrays. **a** Schematic illustration of AMOLED fabrication. **b** Normalized electroluminescence spectrum of the OLED. **c** Current efficiency and power efficiency with brightness characteristic curves. **d** LUMO and HOMO energy level alignment diagram of the device. **e** Transfer and output curves of the OTFT driving the OLED. **f** Switching characteristics of the OTFT repeatedly controlled by the *V*_GS_ signal

To further demonstrate the practical applicability of the high-density OTFT active-matrix arrays, all-organic AMOLED devices were fabricated and characterized using a simplified OTFT–OLED driving configuration as a proof-of-concept demonstration. The fabrication procedures of the OTFT arrays and the AMOLED devices are schematically illustrated in Figs. S12 and 4a, respectively. The construction of the AMOLEDs involved three essential steps: 1) OTFT active-matrix arrays were first integrated on flexible substrates to serve as the driving backplane. 2) a patterned polystyrene-block-poly(ethylene-ran-butylene)-block-polystyrene (SEBS) layer was then introduced as the pixel definition layer (PDL). This layer not only defined the emission area, preventing electrical crosstalk between pixels, but also provided a planarized surface for OLED deposition. 3) OLED emission units and the cathode were thermally evaporated within the PDL-defined regions (aligned with the transistor drain electrodes), thereby integrating the driving circuit with OLED (see the Experimental Section for detail). It should be emphasized that, in preparing the all-organic AMOLED devices described here, Au electrodes of the OTFT arrays were not employed as the OLED anode. Instead, the conductive polymer poly(3,4-ethylenedioxythiophene):polystyrenesulfonate (PEDOT:PSS) was used as the source and drain electrode material. This choice is mainly attributed to the bottom-emitting structure of the OLEDs reported here. A 30 nm Au film exhibits low transmittance in the visible range and cannot meet the optical transparency requirements of a transparent anode. In contrast, PEDOT:PSS not only exhibits a work function (~ 5.1 eV) well-matched with organic semiconductors but also provides superior transparency and mechanical flexibility [23], making it highly compatible with the all-organic AMOLED system. Based on this electrode structure, we achieved stable brightness output from the OLEDs, as shown in Fig. 4b, c. Figure 4b presents the normalized electroluminescence spectrum of the fabricated OLED, with a main emission peak at ~ 560 nm, corresponding to stable yellow light emission. Figure 4c shows the current efficiency (CE)-brightness-power efficiency (PE) characteristic curves of the OLED. At a brightness of 809.9 cd m^−2^, the device delivered excellent photoconversion performance, with maximum CE and maximum PE reaching 25.6 cd A^−1^ and 4.5 lm W^−1^, respectively. The external quantum efficiency (EQE) of the OLED devices is as shown in Fig. S13. This excellent photoconversion performance provides a solid foundation for subsequent integration with OTFTs. The energy-level diagram of the OLED device (Fig. 4d) further reveals that PEDOT:PSS, serving as the anode, forms favorable energy alignment with the organic hole transport layer, effectively lowering the hole injection barrier.

In the integrated AMOLED system (Fig. 4e), the OLED driving current was effectively modulated by the OTFT array via both the gate voltage (*V*_GS_) and data voltage (*V*_DATA_) with the maximum current exceeding 0.1 mA. The transfer characteristics (Fig. S14) confirm excellent switching behavior and a high on/off ratio (~ 10^5^). The devices exhibited a distinct linear regime at low voltages and a saturation regime at high voltages, indicative of efficient charge injection and transport within the channel. Furthermore, repeated on/off cycling for over 100 cycles (Fig. 4f) demonstrated stable switching responses, underscoring reliable operational stability. Collectively, these results demonstrate the successful integration of all-organic OTFT circuits with OLEDs, validating their feasibility for high-resolution displays and paving the way toward next-generation flexible and wearable electronic systems.

**Fig. 5:**
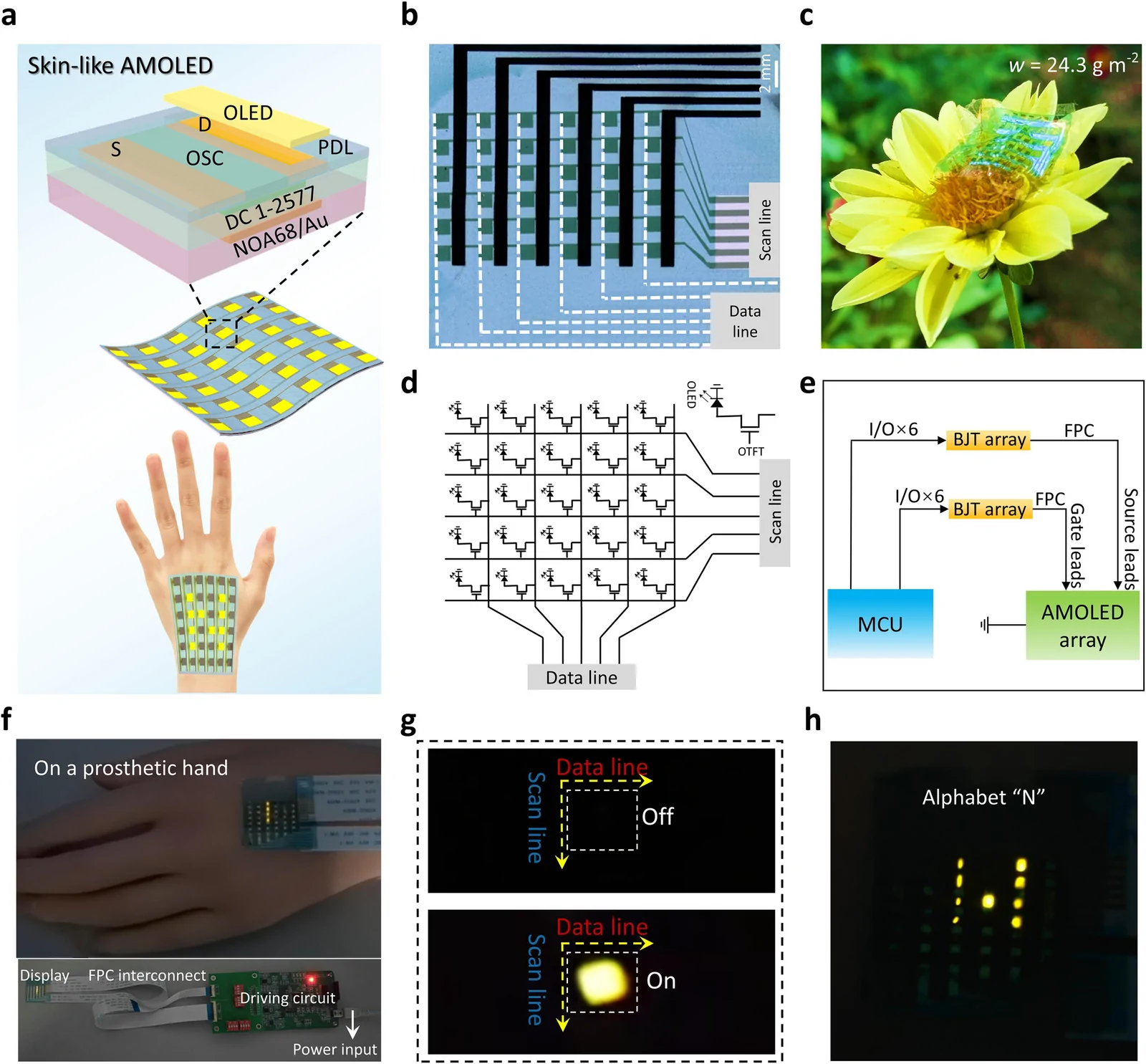
Ultralight and skin-like all-organic AMOLED arrays. **a** Schematic configuration of the skin-conformal AMOLED system. **b** Optical micrograph of a 6 × 6 AMOLED pixel array. **c** Photograph of the ultralight AMOLED array placed on a flower without deformation. **d** Schematic diagram of the AMOLED array control circuit. **e** Schematic diagram showing the connection of AMOLED to printed circuit board (PCB) via flexible printed circuits (FPCs). **f** Photograph of the AMOLED array placed on a prosthetic hand while the array is operated, and the overall photograph of the display system. **g** Photographs showing clear on/off contrast. **h** Demonstration of the device displaying the letter “N”

Beyond all-organic integration at the material and device levels, the fabricated AMOLED arrays also exhibit unique advantages in terms of flexibility and ultralight characteristics. Figure 5a shows a schematic of the skin-like AMOLEDs. Benefiting from the exceptional flexibility of all-organic system provided by NOA68, the devices show excellent compatibility with skin, thereby laying the groundwork for skin-like displays. Figure 5b shows an optical microscope image of a 6 × 6 AMOLED array, where the lead boundaries are clearly defined with a line width and spacing of 500 μm. Such structural precision provides a solid basis for subsequent connection to flexible printed circuits (FPCs) and reliable pixel driving. In addition, the device demonstrates an ultralight weight of 24.3 g m^−2^ (Fig. S15). When placed on the surface of a flower (Fig. 5c), the flower did not deform, highlighting the thin and lightweight nature of the array. The schematic diagrams in Fig. 5d, e illustrate the operating principle of the AMOLED array. Scan lines are connected to the OTFT gates for row addressing, while data lines are connected to the OTFT sources to inject current into the selected pixels. Upon activation of a scan line, the corresponding OTFTs switch on, enabling the data lines to drive current into the OLEDs and thus trigger pixel-level light emission. Figure 5f shows a complete photograph of the display system. Anisotropic conductive film (ACF) was first laminated onto the FPC, which was then precisely aligned and bonded to the OTFT output terminal to establish a stable electrical connection. Subsequently, FPC wires were connected to the drive circuit board, and stable light emission was achieved under an external power supply. In addition, the device can still emit light when connected to a prosthetic hand, proving that it has good mechanical stability. Clear on/off contrast was also observed in real photographs (Fig. 5g), intuitively demonstrating effective pixel switching from dark to bright states. Finally, Fig. 5h shows the device displaying the letter “N”. In this work, the AMOLED array is presented as a proof-of-concept demonstration to validate the compatibility of the proposed full-photolithographic transistor array with OLED integration. The current array resolution is intentionally maintained at a relatively modest level to ensure reliable fabrication and compatibility with flexible substrates. With further improvements in device fabrication and interconnection processes, higher-density AMOLED arrays are expected to be achievable in future studies. These results showcase an all-organic AMOLED system that integrates outstanding flexibility, ultralight weight, and reliable pixel-driving capability. This work provides a scalable integration strategy for all-organic displays and underscores their strong potential for next-generation wearable and skin-conformal electronic systems.

## Conclusions

In summary, we have established a full-photolithographic strategy for fabricating high-density OTFT active-matrix arrays that simultaneously overcomes long-standing challenges in organic semiconductor patterning and large-area integration. By combining “synergistic interfacial modulation” and a high-precision semiconductor micropatterning approach, the arrays exhibit both excellent electrical performance and remarkable device uniformity, achieving record integration densities. Beyond the transistor arrays themselves, their practical applicability was validated through integration with OLEDs to realize all-organic AMOLED arrays, which feature stable emission, ultralight weight, and conformability to complex surfaces. This work provides a versatile and scalable pathway that effectively bridges advanced micro/nano fabrication techniques with organic semiconductor technology. The methodology is fully compatible with large-area manufacturing and can be broadly extended to diverse classes of organic electronic systems. Looking forward, this approach holds great promise for enabling high-resolution flexible displays, skin-conformal electronics, and next-generation wearable platforms, and may provide a solid technological foundation for the industrial-scale deployment of organic thin-film transistor technologies.

## Supplementary Information

Below is the link to the electronic supplementary material.

Supplementary file1 (DOCX 35863 KB)
