# Supplementary material for: Full-Photolithographic High-Density Skin-Like Transistor Arrays for All-Organic Active-Matrix Displays
Source: Nanomicro Lett. 2026 Mar 4;18:270. doi: 10.1007/s40820-026-02107-w (PMC12960907; doi:10.1007/s40820-026-02107-w)
Supplement: Supplementary file 1 — Supplementary file1 (DOCX 35863 KB) [file 40820_2026_2107_MOESM1_ESM.docx]

**Full-photolithographic high-density skin-like transistor arrays for all-organic active-matrix displays**

Peng Xue, Juntong Li, Xiaoli Zhao*, Yanping Ni, Hongyan Yu, Xianghui Liu, Bowen Xiang, Yao Fu, Junru Zhang, Pengbo Xi, Baoying Sun, Xiang Song, Yijun Shi, Guodong Zhao, Mingxin Zhang, Yanhong Tong, Qingxin Tang*, and Yichun Liu

Center for State Key Laboratory of Integrated Optoelectronics, and Key Lab of UV-Emitting Materials and Technology of Ministry of Education, College of Physics, Northeast Normal University, 5268 Renmin Street, Changchun 130024, China

*Corresponding author. E-mail addresses: tangqx@nenu.edu.cn (Q. Tang), zhaoxl326@nenu.edu.cn (X. Zhao)


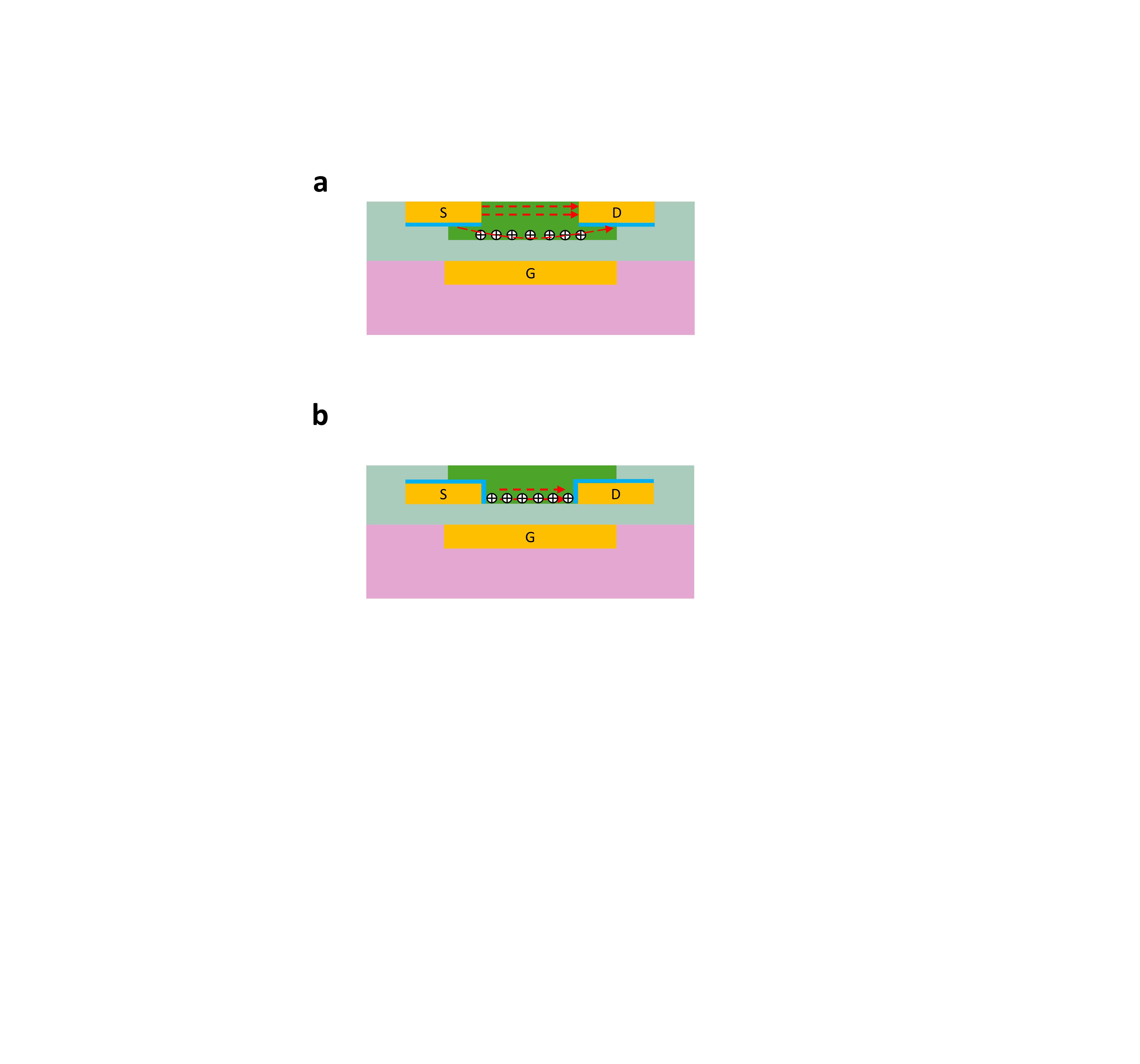


**Figure S1**. Schematic illustrations of transistors with different configurations. (a) Bottom-gate top-contact structure. (b) Bottom-gate bottom-contact structure. In the top-contact configuration, the larger charge injection area between electrodes and semiconductor facilitates a larger effective contact area between the electrodes and the semiconductor, which is beneficial for charge injection.





**Figure S2.** (a) Atomic force microscopy (AFM) images of organic semiconductor films in the pristine state, after patterning with a dual-protective layer, and after patterning without a protective layer. (b) Transfer characteristics of transistors fabricated on organic semiconductor films under different processing conditions. (c) Statistical comparison of field-effect mobility extracted from multiple devices.


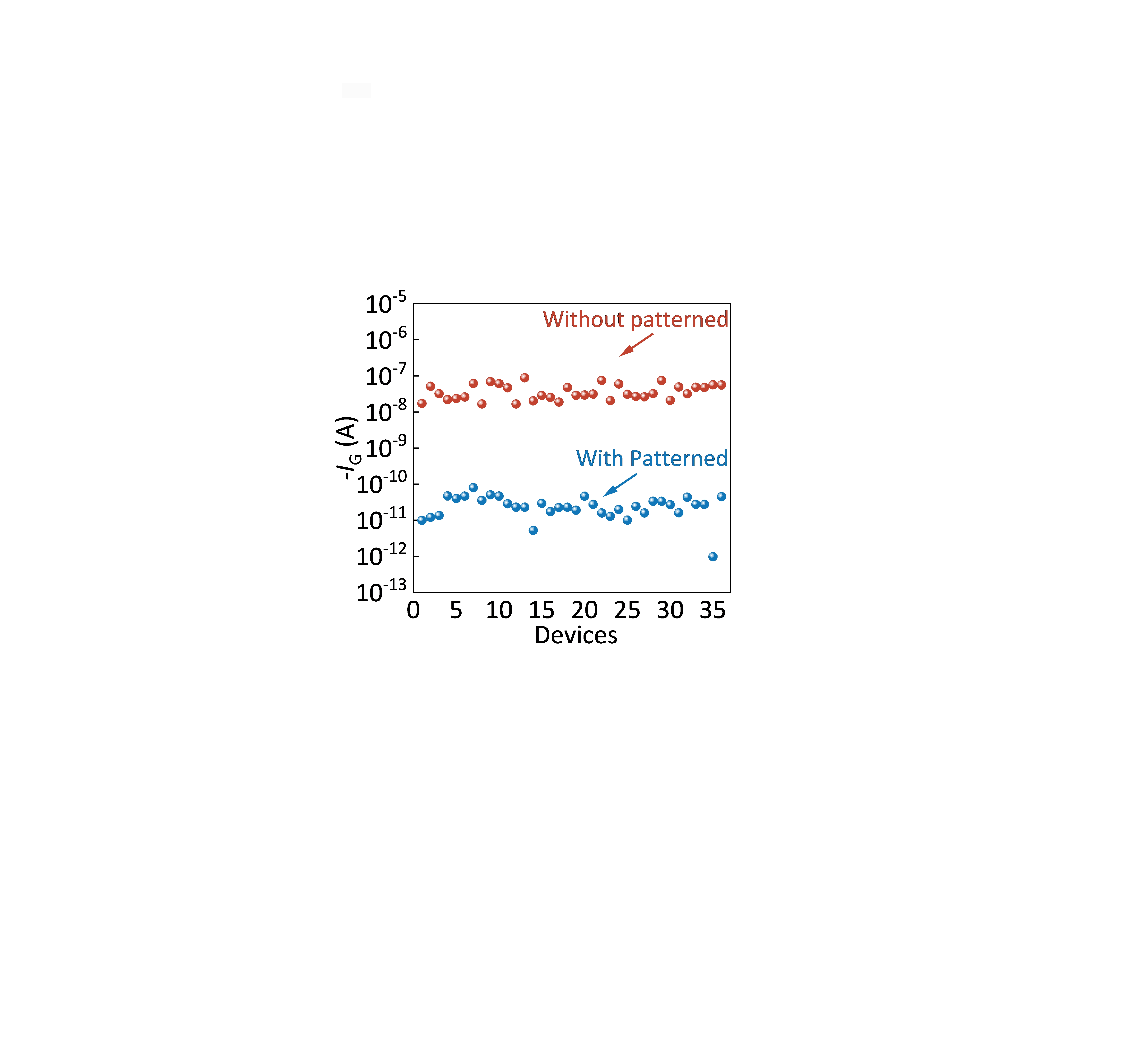


**Figure S3.** Comparison of leakage current characteristics of OTFTs fabricated with and without the dual-protective-layer photolithography process. Patterned semiconductors effectively suppress transistor leakage current and supporting the electrical stability of high-density integrated arrays.


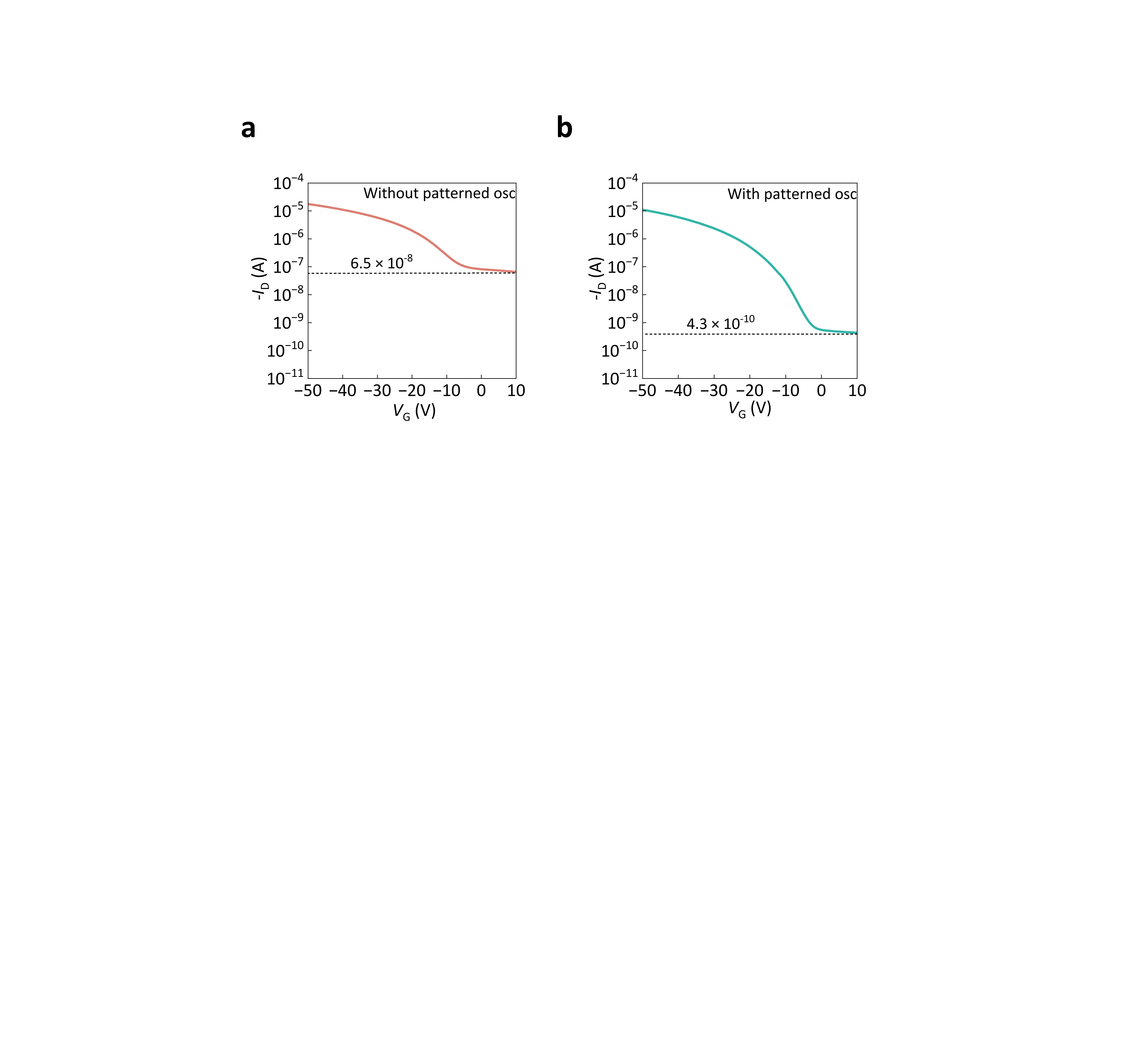


**Figure S4.** a) Transfer curve of the unpatterned semiconductor device. b) Transfer curve of the patterned semiconductor device. Precisely patterned semiconductors can minimize crosstalk between adjacent pixels, reduce the device’s off-state current, and thereby enabling independent switching and effective signal addressing of the device.







**Figure S5.** Schematic diagram comparing existing photolithographic processes for organic semiconductors with the photolithographic process employing a dual-protection.


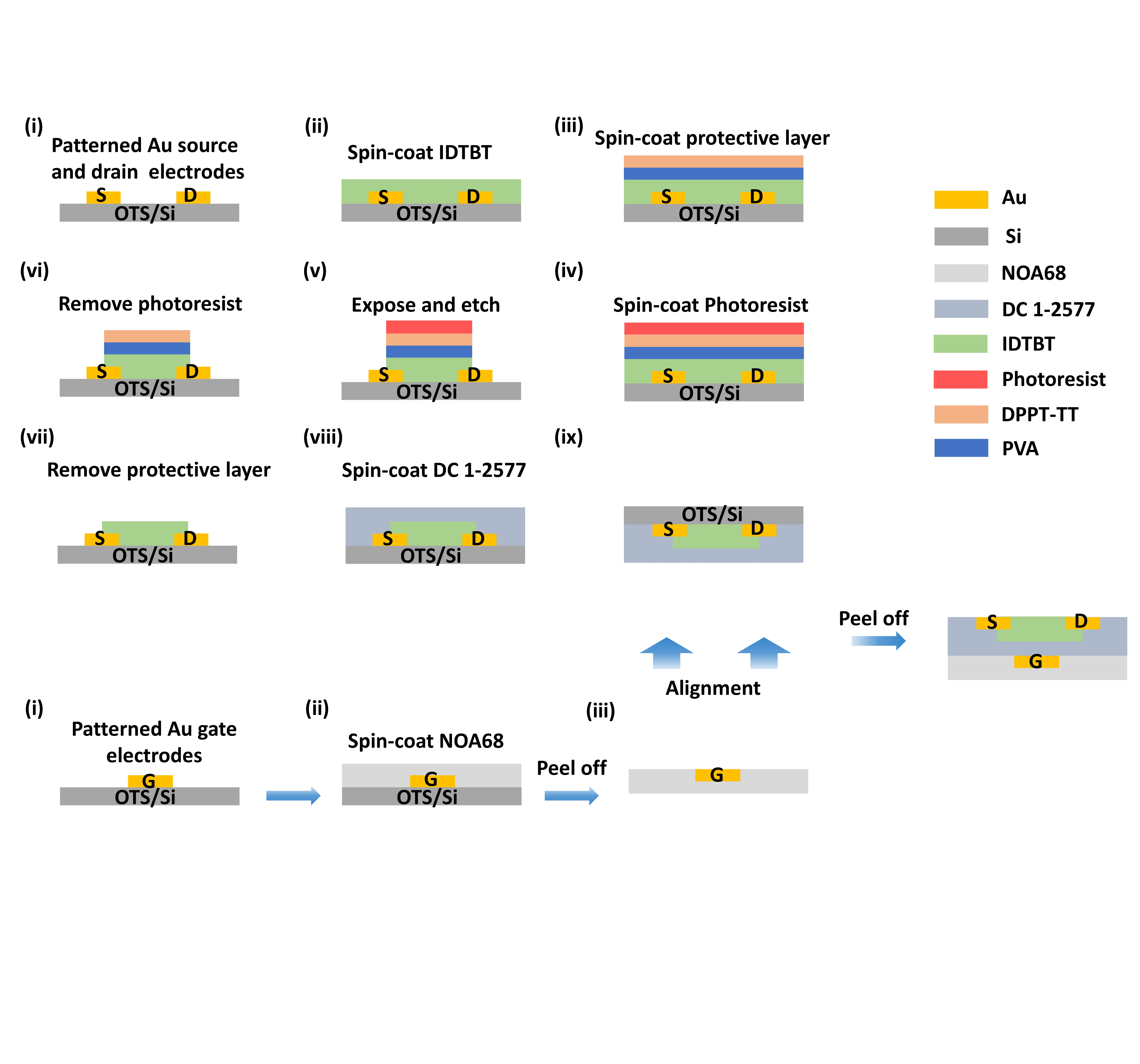


**Figure S6.** Schematic illustration of the full-photolithographic fabrication process for skin-like OTFT active-matrix arrays.


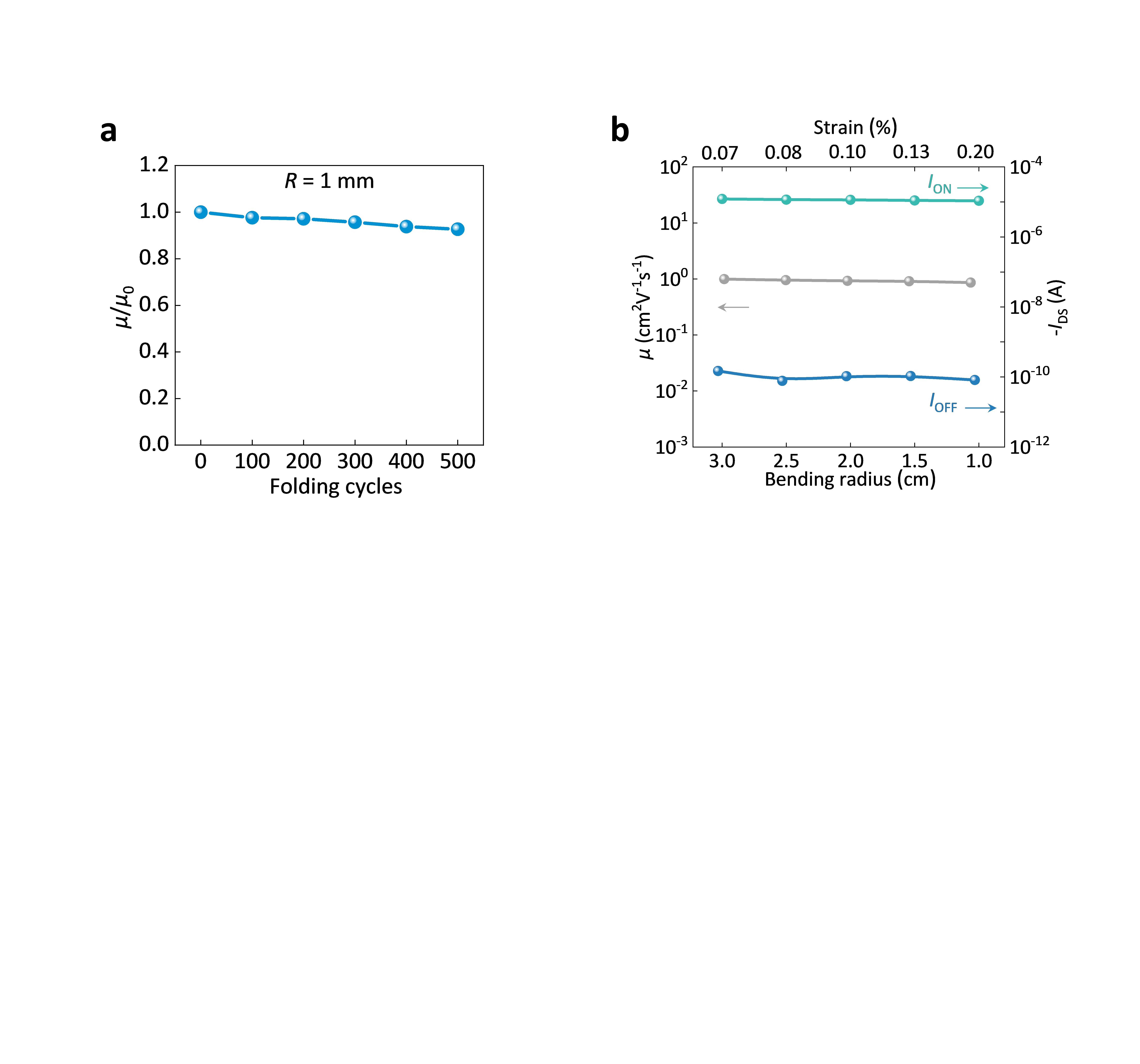


**Figure S7.** (a) The functional relationship between the folding cycles and the normalized mobility. (b) The functional relationship between the strain and the electrical properties of OTFT.


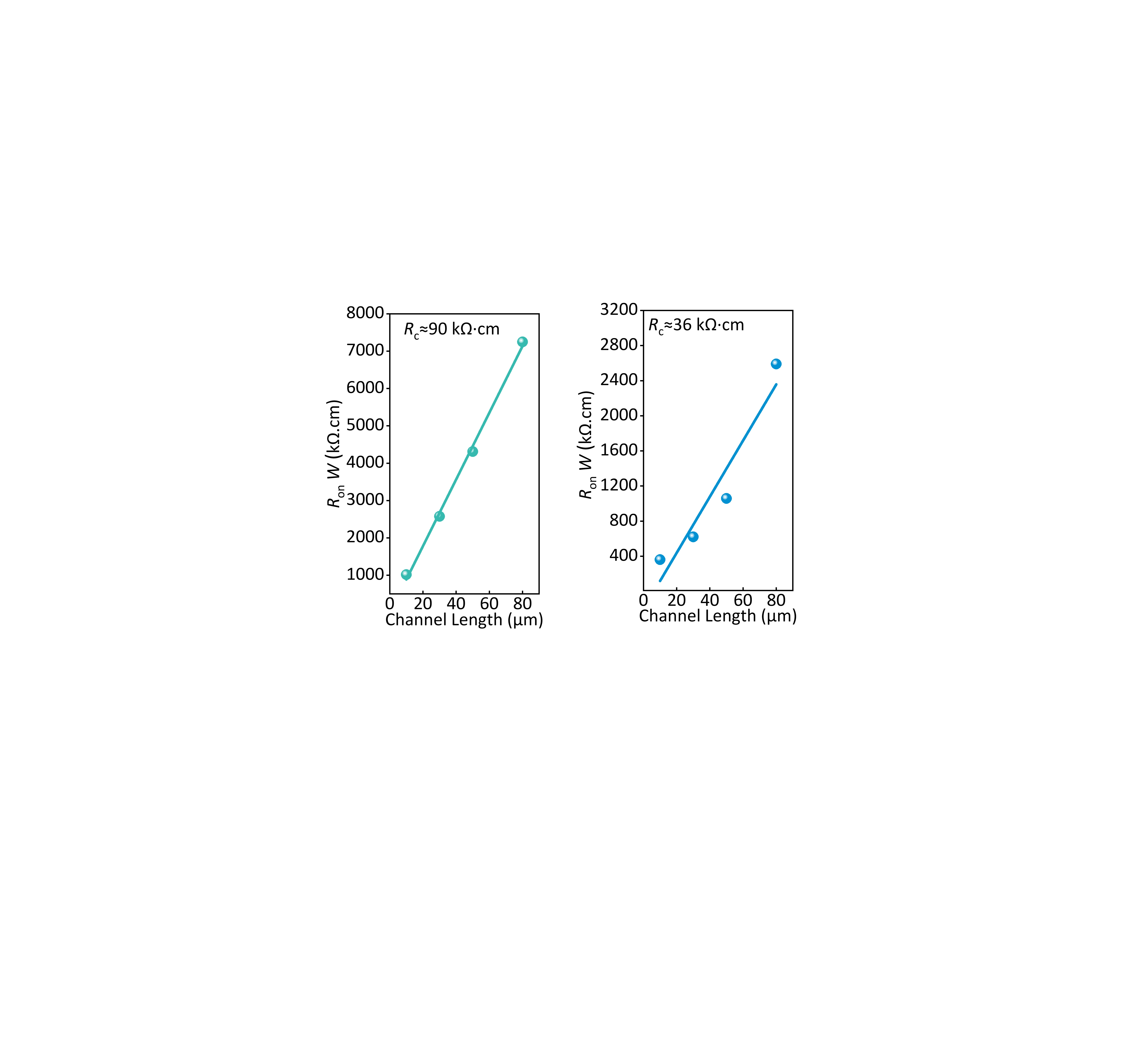


**Figure S8.** Contact resistance extracted by the transmission line method (TLM) for DC 1-2577-based devices before and after PFBT modification (*V*_G_ = -18 V, *V*_D_ = -1 V).


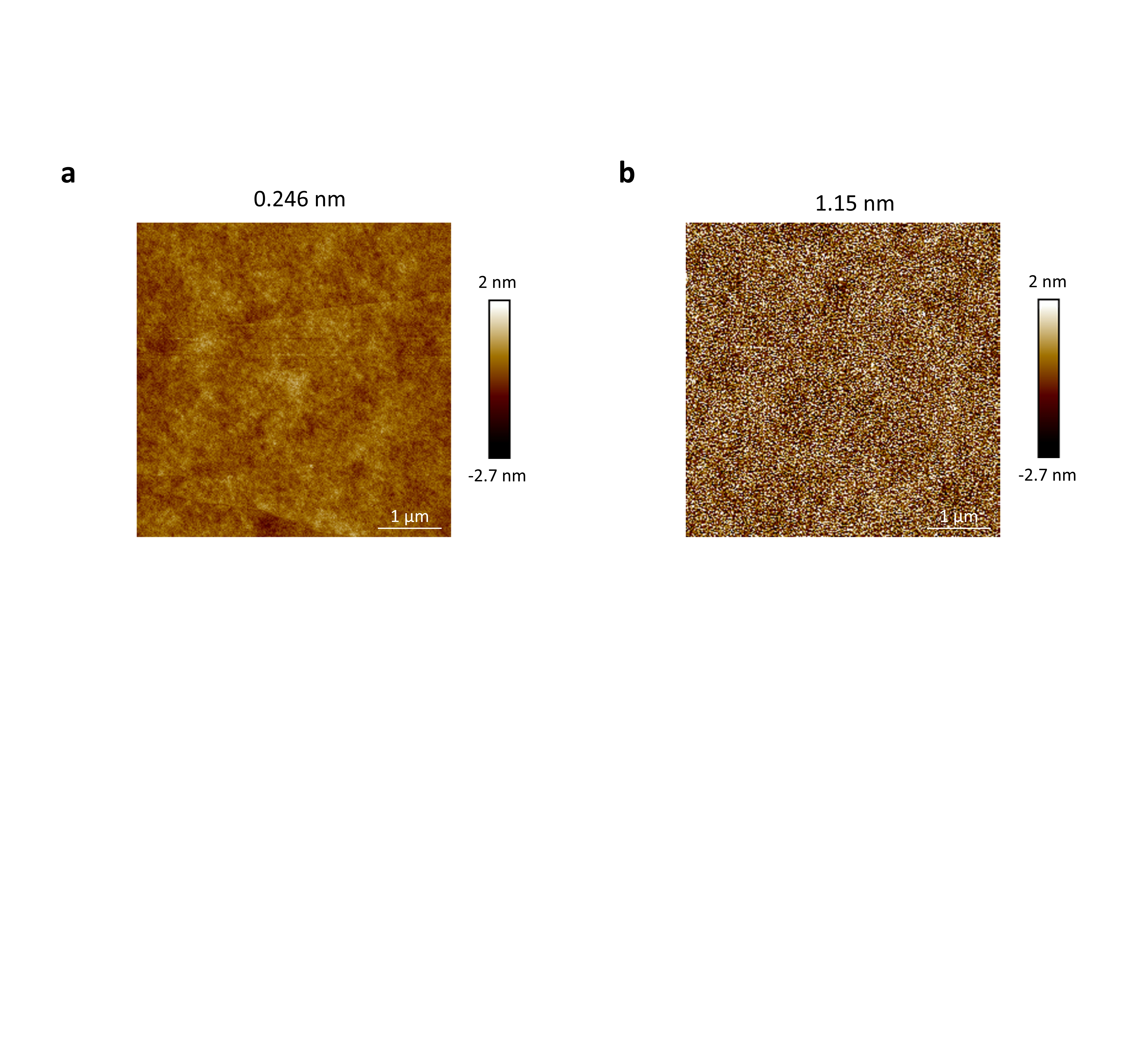


**Figure S9.** Surface morphology and roughness evolution of PMMA dielectric films before and after oxygen plasma treatment.


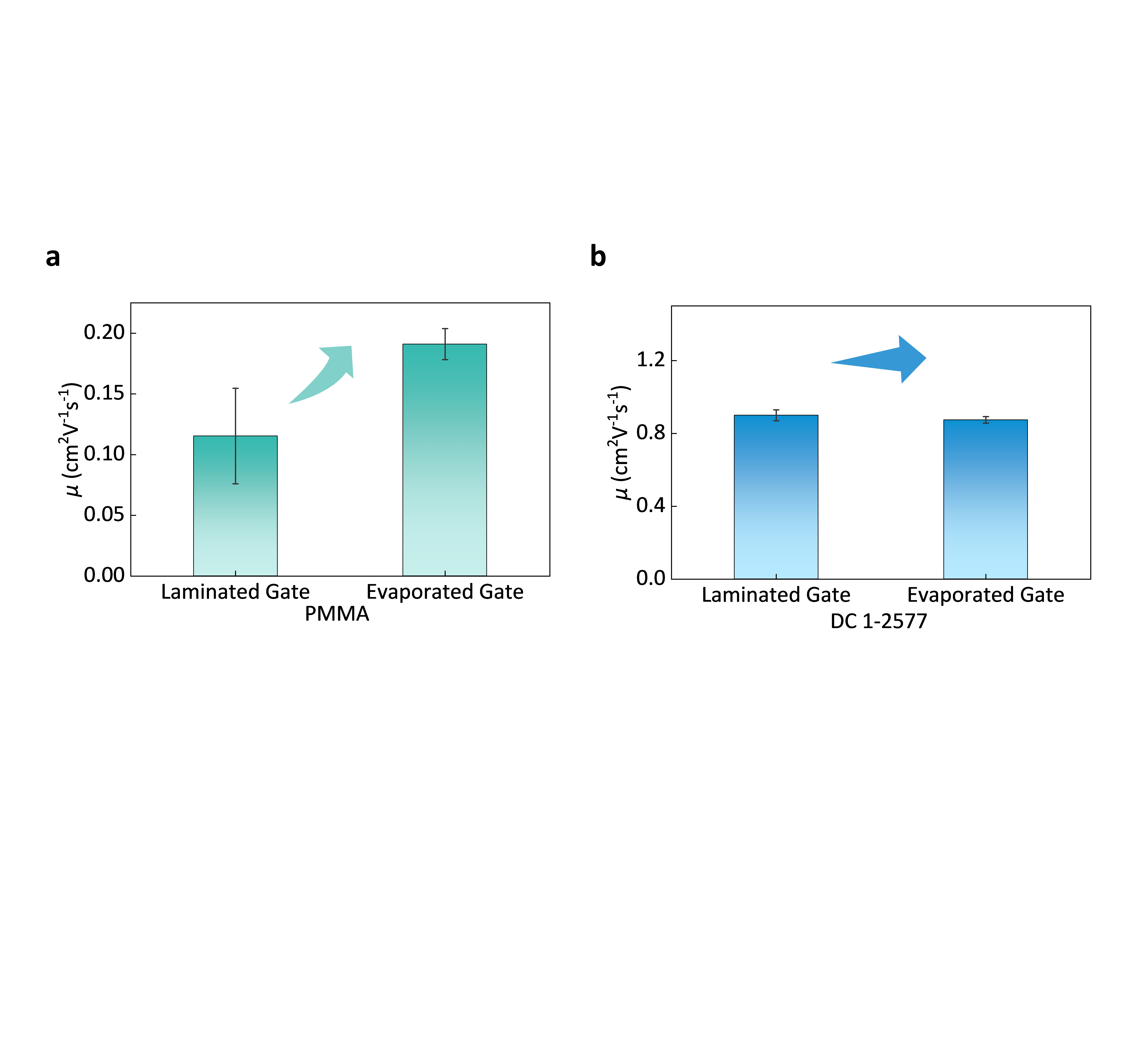


**Figure S10.** (a) The mobility comparison of laminated gate and evaporated gate structure devices with PMMA as the dielectric layer. (b) The mobility comparison of laminated gate and evaporated gate structure devices with DC 1-2577 as the dielectric layer.


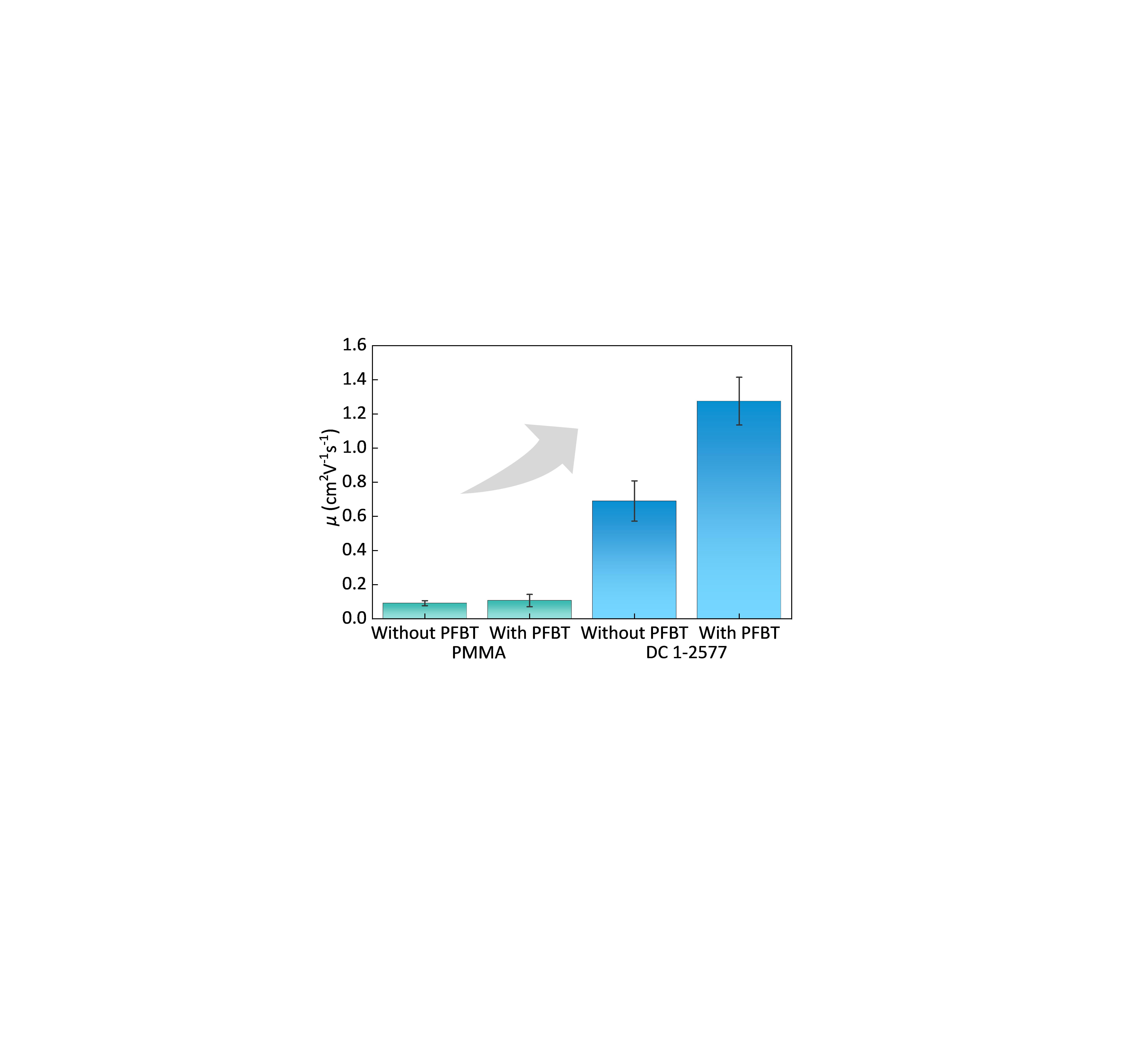


**Figure S11.** Comparison of field-effect mobilities for four types of devices with different combinations of dielectric layers (PMMA or DC 1-2577) and electrode modification (with or without PFBT).


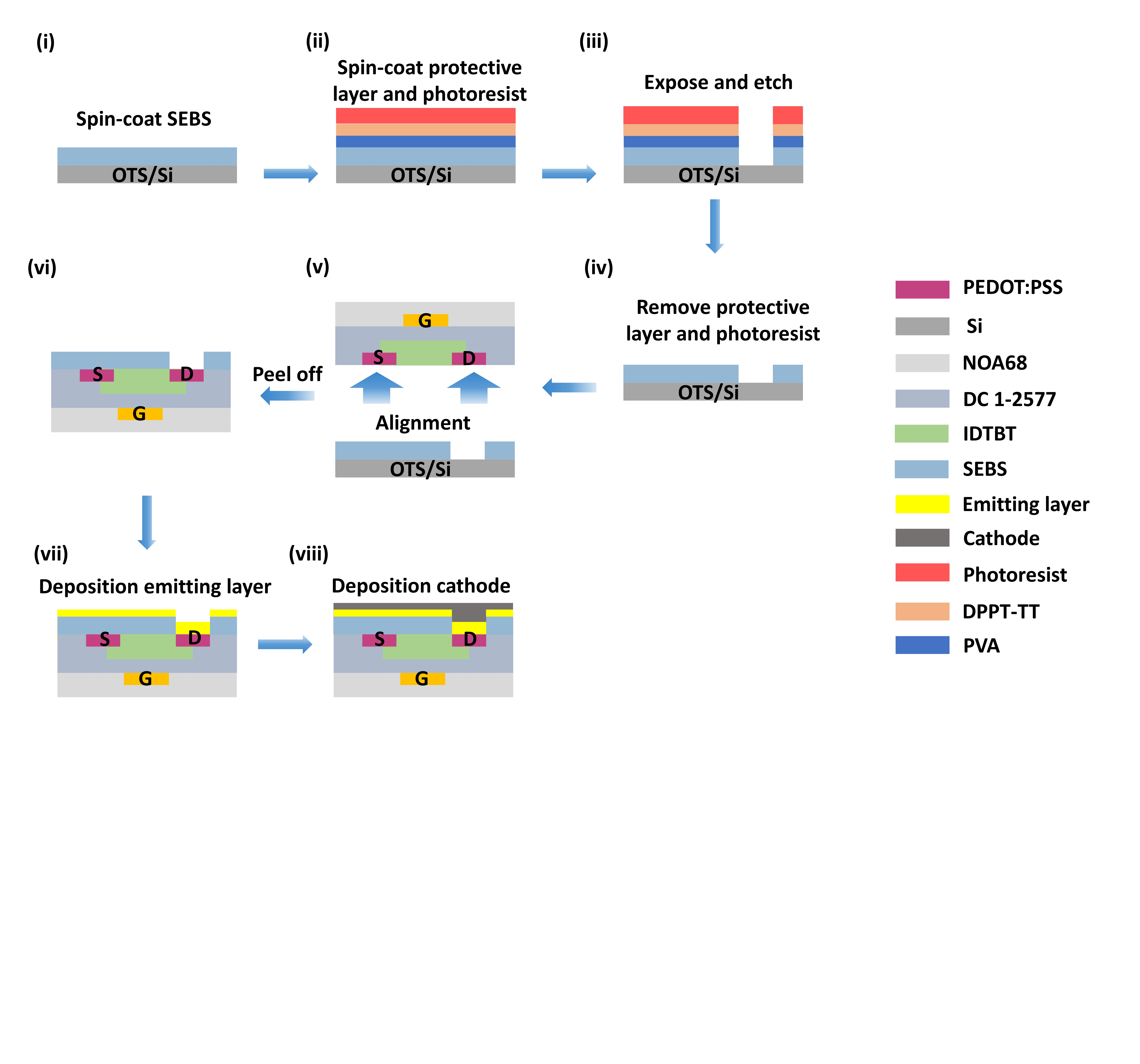


**Figure S12.** Schematic illustration of the fabrication procedures for all-organic AMOLED devices.


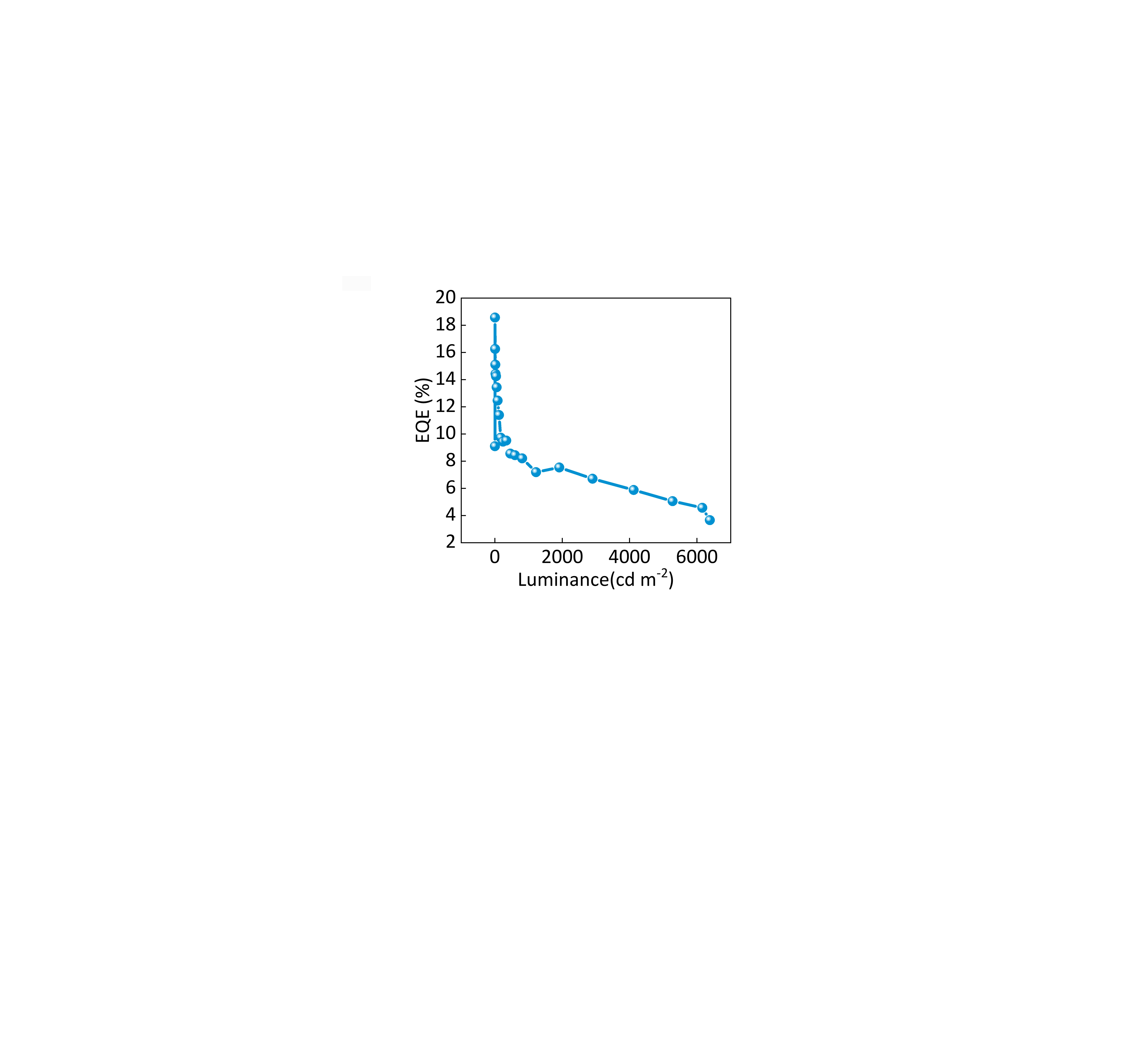


**Figure S13.** Curve of EQE versus luminance for the OLED.


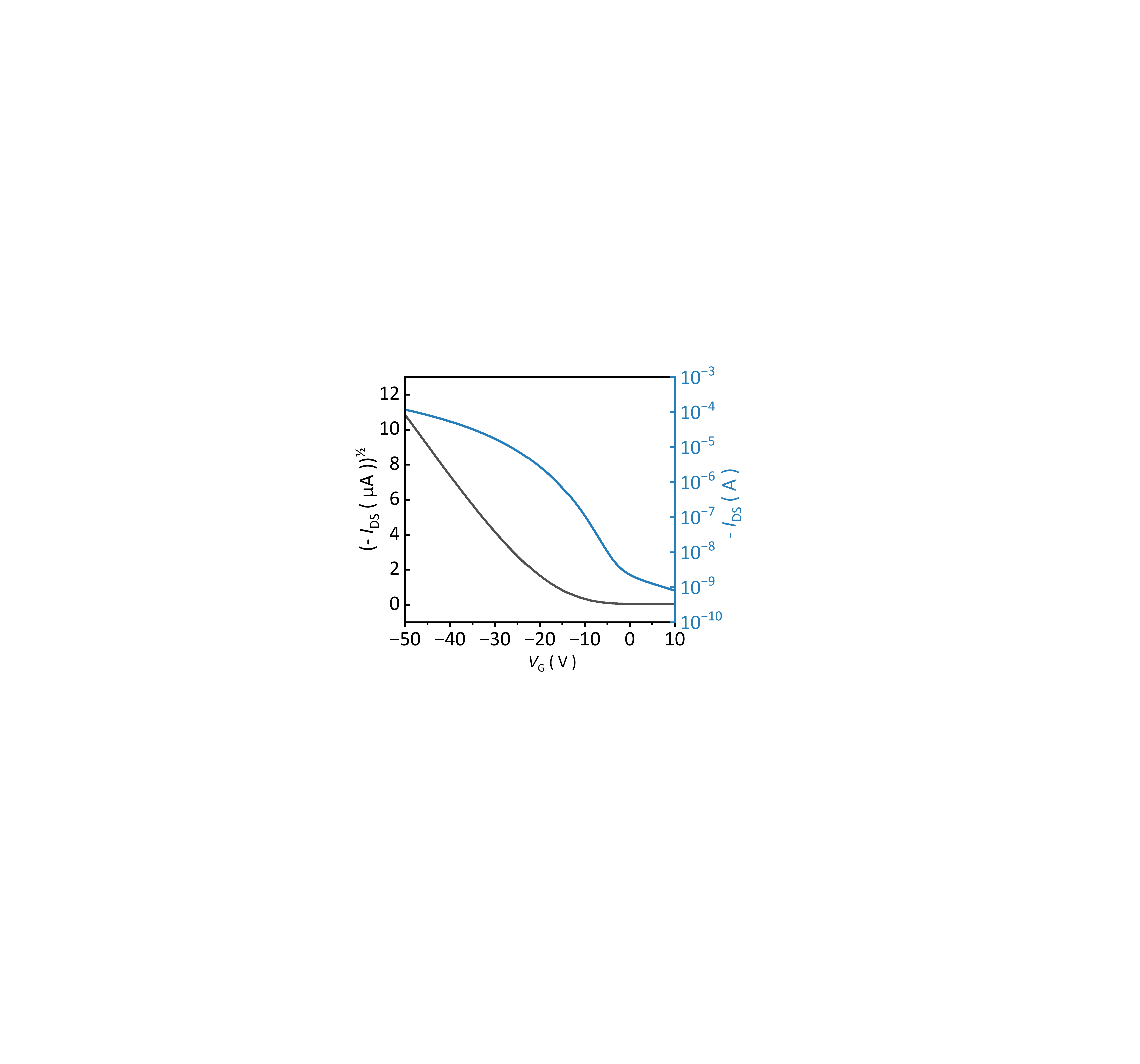


**Figure S14.** Transfer characteristics of OTFTs in all-organic AMOLED arrays.


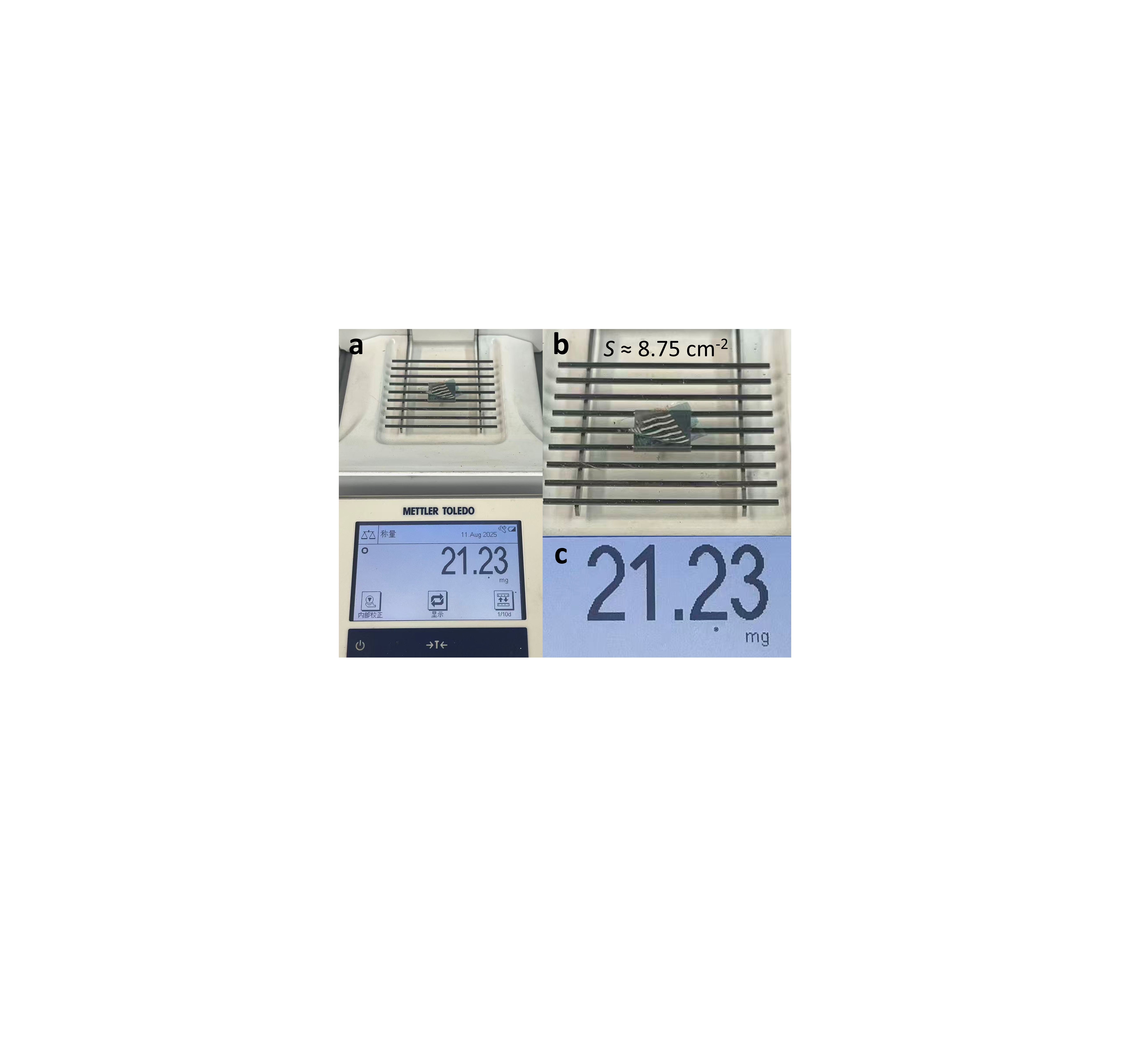


**Figure S15.** a) Photograph of the weight measurement for all-organic AMOLED array. b) The 2.5 × 3.5 cm^2^ AMOLED array was placed on the measuring table of an electronic balance. c) The test weight of the whole device is 21.23 mg.

| Device type | Device density (devices cm^-2^) | *μ* (cm^2^V^-1^s^-1^) | Reference |
| --- | --- | --- | --- |
| Flexible | 1.6 | 0.002-0.1 | [[1](#_ENREF_47)] |
| Flexible | 4 | 0.88 | [[2](#_ENREF_48)] |
| Flexible | 7 | 0.9 | [[3](#_ENREF_49)] |
| Flexible | 16 | 0.35 | [[4](#_ENREF_50)] |
| Flexible | 347 | 1.11 | [[5](#_ENREF_51)] |
| Flexible | 375 | 1.08 | [[6](#_ENREF_52)] |
| Flexible | 1560 | 1-2 | [[7](#_ENREF_18)] |
| Flexible | 62500 | 1.13 | This work |

**Table S1** Comparison of device densities in reported organic transistor active-matrix arrays.

**References**

1. J. A. Rogers, Z. Bao, K. Baldwin, A. Dodabalapur, B. Crone et al., Paper-like electronic displays: Large-area rubber-stamped plastic sheets of electronics and microencapsulated electrophoretic inks. Proc. Natl. Acad. Sci. **98**(9), 4835-4840, (2001). <https://doi.org/10.1073/pnas.091588098>
2. K. Fukuda, Y. Takeda, Y. Yoshimura, R. Shiwaku, L. T. Tran et al., Fully-printed high-performance organic thin-film transistors and circuitry on one-micron-thick polymer films. Nat. commun. **5**, 4147, (2014). <https://doi.org/10.1038/ncomms5147>
3. K. Fukuda, Y. Takeda, M. Mizukami, D. Kumaki & S. Tokito, Fully solution-processed flexible organic thin film transistor arrays with high mobility and exceptional uniformity. Sci. Rep. **4**, 3947, (2014). <https://doi.org/10.1038/srep03947>
4. E. K. Lee, C. H. Park, J. Lee, H. R. Lee, C. Yang et al., Chemically robust ambipolar organic transistor array directly patterned by photolithography. Adv. Mater. **29**(11), 1605282, (2017). https://doi.org/10.1002/adma.201605282
5. S. Wang, J. Xu, W. Wang, G.-J. N. Wang, R. Rastak et al., Skin electronics from scalable fabrication of an intrinsically stretchable transistor array. Nature. **555**(7694), 83-88, (2018). https://doi.org/10.1038/nature25494
6. H. Ren, J. Zhang, Y. Tong, J. Zhang, X. Zhao et al., Synchronously improved stretchability and mobility by tuning the molecular weight for intrinsically stretchable transistors. J Mater Chem C. **8**(44), 15646-15654, (2020). https://doi.org/10.1039/D0TC02363A
7. X. Zhao, S. Wang, Y. Ni, Y. Tong, Q. Tang et al., High‐performance full‐photolithographic top‐contact conformable organic transistors for soft electronics. Adv. Sci. **8**(9), 2004050, (2021). https://doi.org/10.1002/advs.202004050
